# Initial Data Analysis for Cancer Registries: A Structured Framework and Demonstration Using Slovenian Cancer Registry Data

**DOI:** 10.3390/cancers18142332

**Published:** 2026-07-20

**Authors:** Maja Jurtela, Lara Lusa, Tina Žagar, Nika Bric, Mojca Birk, Vesna Zadnik

**Affiliations:** 1 Slovenian Cancer Registry, Institute of Oncology Ljubljana, 1000 Ljubljana, Slovenia; 2 Faculty of Medicine, University of Ljubljana, 1000 Ljubljana, Slovenia; 3 Department of Mathematics, Faculty of Mathematics, Natural Sciences and Information Technologies, University of Primorska, 6000 Koper, Slovenia; 4 Institute for Biostatistics and Medical Informatics, Faculty of Medicine, University of Ljubljana, 1000 Ljubljana, Slovenia

**Keywords:** initial data analysis, cancer registry, metadata, reproducibility, traceability, transparency, cancer informatics, real-world data, secondary data use, FAIR data

## Abstract

Background/Objectives: Initial data analysis (IDA) is essential for valid and reproducible statistical analyses, but existing IDA frameworks were primarily developed for single-study datasets. Cancer registries (CRs) are extensive data systems characterized by continuous updates, repeated data extraction, multiple analytical uses, and evolving classification systems, which create requirements not addressed by existing IDA frameworks. This study aims to develop a structured IDA framework adapted to CRs. Methods: We conceptualized IDA in CRs as a process spanning three data states: operational registry data, the extracted dataset and the analysis-ready dataset. The framework was developed by adapting existing IDA principles to the CR setting and organizing them into four stages: metadata, cleaning, screening, and reporting. The approach is based on predefined and versioned rule sets, structured recording of data processing, and metadata-based linkage between dataset definitions, data cleaning rules, screening outputs, the final report and dataset. A demonstrative survival dataset from the Slovenian Cancer Registry was used to illustrate implementation. Results: The framework is represented by the item set defining the activities and expected outputs of the structured IDA process in CRs. In the use case, metadata specified the dataset scope, intended use, variables, coding context, and applicable rules. Execution of the selected rules produced an analysis-ready dataset with traceable data cleaning steps, documented eligibility decisions, screening outputs describing the general and analysis-specific data properties, and the IDA report intended for external researchers to be delivered alongside the data. Conclusions: The proposed framework extends existing IDA approaches to meet the specific requirements of CRs. It supports consistent dataset preparation and with that improves transparent and reproducible data use.

## Simple Summary

Cancer registries collect and maintain extensive amounts of data about people diagnosed with cancer. They are essential for monitoring cancer burden and supporting public health decisions. However, before registry data can be used for analysis, many decisions must be made about which records to include, how variables are defined, how data are checked, and how final datasets are documented. If these steps are not performed systematically and transparently, results may be unreliable and difficult to reproduce. This study proposes a structured framework for preparing cancer registry data before statistical analysis. Using data from the Slovenian Cancer Registry, we show how metadata, predefined rules, screening outputs, and reports can support more consistent, traceable, and reproducible use of registry data.

## 1. Introduction

Initial data analysis (IDA) comprises all activities after data collection and before final statistical analysis. It is critical for the reproducibility and validity of analyses, because choices made while preparing data can affect the results obtained from the same underlying data [1,2]. The term IDA has been established in the statistical literature [3] yet a structured and transparently reported approach to IDA is still not routinely done. A structured framework for single-study observational data was proposed by the STRATOS (STRengthening Analytical Thinking for Observational Studies) initiative, consisting of metadata preparation, data cleaning, data screening, initial data reporting, refinement of the analysis plan, and reporting of IDA in scientific papers [4]. In the present work, we focus on the first four components, which directly support the preparation and documentation of analysis-ready datasets. Subsequent work adapted these principles to specific analytical settings, including regression [5] and longitudinal studies [6], focusing mostly on data screening.

However, the increasing use of routinely collected health data has shifted attention towards complex datasets, such as those from cancer registries (CRs). Population-based CRs are long-established systems present worldwide that systematically collect longitudinal data on cancer patients, integrating information from multiple sources. CRs enable both primary uses of data (e.g., routine reporting of cancer burden) and secondary uses (e.g., epidemiological research) and are central to public health monitoring [7,8]. Survival analysis is one of the key analytical uses of CR data. While international organizations such as the International Association of Cancer Registries (IACR) and the European Network of Cancer Registries (ENCR) provide quality standards and validation tools, these primarily focus on data quality assurance rather than on the full process of data preparation and interpretation [9,10,11]. Large international initiatives such as the CONCORD programme further emphasize the importance of standardized analytical datasets and procedures to make results comparable across registries, countries and time periods [12].

There are several reasons why existing IDA frameworks require adaptation for CRs. General IDA frameworks are primarily developed for single-study observational datasets where data collection is completed before analysis begins and the dataset is usually already defined by the study design [4,5,6]. CRs, in contrast, operate as continuously updated information systems and data are repeatedly extracted from a live database, transformed for different uses, and interpreted in relation to changing coding systems and validation procedures, reflecting the life-cycle logic of register-based statistics [13,14]. Dataset identity is therefore not self-evident and further complicated by a high variety of uses of CR data such as incidence reporting, research datasets or internal monitoring. At the same time, these dataset-generating decisions also need to be transparent. If a structured mechanism to separate rule definition from rule execution is not in place, most commonly in the form of structured metadata, these processes are hidden in informal documentation, programming code, or analyst-specific decisions. The problem is compounded by the dynamic rule context of CRs. Cancer registry data rely on evolving classification systems, such as the International Classification of Diseases for Oncology (ICD-O) for tumour topography and morphology, and the TNM Classification of Malignant Tumours for cancer staging [15,16], changing internal coding practices, and updates in validation tools; as a result, the same record may be interpreted differently depending on the rule versions [17,18]. Existing general IDA frameworks assume stable definitions and do not require a declaration of rule versions, which in CR would lead to a loss of comparability across time and between uses of data. Additionally, although existing quality-assurance procedures [19,20] and guidelines for CRs address important aspects, such as completeness, consistency, and validity [8,21,22], they overlook the broader role of IDA since they do not provide a structure linking its activities to reproducible dataset generation. At the same time, CRs share enough common ground, such as core variables, disease classifications, and recurring reporting contexts, while still facing comparability challenges across registries and time periods, to justify a domain-specific IDA framework [23].

The contribution of this article is therefore twofold: first, it identifies why existing IDA frameworks require adaptation for CR data; second, it proposes a structured IDA framework for CRs. To our knowledge, this is the first structured IDA framework developed for CRs.

## 2. Materials and Methods

### 2.1. Conceptual Development of the Structured Initial Data Analysis Framework for Cancer Registries

The starting point for the development of the proposed framework was the STRATOS IDA framework for observational studies, in which IDA includes metadata preparation, data cleaning, data screening, and reporting of findings before formal statistical analysis begins [5,6,20]. The adaptation focused on properties of CR data that are not fully addressed in general IDA frameworks. Continuous registry operation and repeated extraction of data for different purposes were addressed by conceptualizing IDA in CRs as a process operating across three dataset states: (A) the operational registry, a continuously updated database for real-time data coding, corrections, and maintenance; (B) the extracted dataset, defined at a specific time point by extraction criteria for a particular analytical purpose; and (C) the final analysis-ready dataset [13,14]. IDA spans the transitions A→B and B→C, with the latter representing its core.

The framework proposed in this paper retains the general logic of the STRATOS IDA framework while adapting it to the operational characteristics of CRs. Table 1 summarizes the aspects of the STRATOS framework that require registry-specific operationalizations, together with the adaptations introduced in the proposed framework. Compared with previous STRATOS-based work, the main registry-specific extensions are therefore not the creation of new IDA stages, but the operationalization of metadata, rule-based preparation, cleaning, screening, reporting, versioning, and locking for reproducible registry dataset generation.

### 2.2. Developing the Practical Approach

To translate the conceptual framework into a usable procedure, we organized the process into a set of items that span four stages: metadata, cleaning, screening, and reporting. Metadata preparation has the purpose of controlling both transitions, A→B and B→C. The cleaning stage executes the transition B→C by applying the rules specified by the metadata. Screening evaluates this transition by characterizing the dataset. Finally, reporting completes it by formalizing dataset locking and documentation. Each stage is divided into items. Each item specifies the activity to be performed, the expected output, and an example of its relevance in the CR context. The item set was developed by mapping the adapted IDA stages to the practical decisions, procedures, and outputs required for dataset generation in registries.

The operationalization distinguishes between definitional metadata and statistical metadata. Definitional metadata are specified before processing to specify the dataset scope, analytical purpose, population, unit of analysis, variables, coding systems, rule context, rule sets, and versioning structure. Statistical metadata are generated during data processing and comprise structured outputs of the IDA process: rule execution logs and summaries, screening summaries, and locking metadata. Definitional metadata describe what the dataset and IDA process are intended to be, whereas statistical metadata record what was done and what resulted from processing. For example, the specification that records with non-positive follow-up time are ineligible is definitional metadata, while the number of records flagged and excluded by that rule is statistical metadata. In the present proposed framework, we therefore use the term “statistical metadata” in a narrow, operational sense. This distinction is consistent with broader statistical information models, such as the Generic Statistical Information Model, which formalizes statistical information objects used as inputs and outputs of statistical production [24].

The core of the implementation was developed through two catalogues: cleaning, understood here as a rule-based data-preparation catalogue, and screening catalogue, to separate documentation stored in metadata from its later execution. We retain the term “cleaning” for consistency with IDA terminology, but use it as an umbrella term for rule-based data preparation in the cancer-registry context. The cleaning rules were grouped into eligibility (inclusion of records), validity (data format, validity and consistency), and data transformation and derivation rules (generating analysis variables such as follow-up time and event status; transformation of the structure and linkage to external data). Screening outputs further characterize the dataset through summaries guided by the intended analytical use. Following previous STRATOS work, structural variables were defined as variables that organize screening summaries across relevant subgroups [5]. Screening was then organized into domains aligned with the intended analytical use of the dataset: population definition, missing data, univariate descriptions, multivariate descriptions, and analysis-specific aspects. All generated outputs were linked through a dataset version identifier: the final locked dataset, rules that produced it, summaries of the process, screening outputs, and the IDA report—the human-readable output of the process. Implementation was carried out in R version 4.4.0 (R Foundation for Statistical Computing, Vienna, Austria) [25], with structured metadata tables imported into the workflow as tabular inputs.

### 2.3. Demonstrative Use Case—Survival Dataset

The applicability of the framework was demonstrated using a lung cancer survival dataset constructed from the Slovenian Cancer Registry (SCR) and included adult lung cancer cases diagnosed between 2011 and 2020, restricted to the first primary tumour per patient. A standard survival dataset demonstrated in this article is intended for time-to-event analysis, using follow-up time and event status to estimate survival probability at several time points since diagnosis; we do not address more complex time-varying covariates, repeated events, or competing events. The intended use of the dataset is thus population-based observed survival estimation across subgroups (sex, 10-year age groups and diagnosis year as structural variables). A dataset was created by enabling a total of 25 rules from the rules catalogue. Supplementary File S2 provides the complete IDA report, while the principal outputs and findings required to understand the implementation are summarized in the article. As one example of survival-specific screening, we assessed whether subgroup-specific k-year survival estimates were supported by sufficient data. For each age subgroup, support was evaluated using two predefined criteria: at least 10 cases with sufficient potential follow-up for the selected time point and at least 10 individuals remaining at risk at that time point. The threshold was used as a pragmatic screening criterion and can be adjusted according to the analytical purpose, outcome frequency, reporting requirements, and acceptable level of uncertainty. These values follow the broader principle that registry estimates based on small cell counts can be unstable and should be reported cautiously [26,27].

## 3. Results

### 3.1. The Structured Initial Data Analysis Framework for Cancer Registries

The proposed IDA framework for CRs defines IDA as a structured process beginning with operational CR data (data state A), proceeding through an extracted dataset (data state B) and ending with a documented analysis-ready dataset (data state C). The framework is represented by an item set that is divided into four stages: metadata, cleaning, screening, and reporting, adapted from the general IDA framework by Huebner et al. [4] and adapted to a registry context. Table 2 summarizes each stage’s purpose, main activities, and outputs. Each stage is further represented by 6–10 items, detailed in Supplementary File S1. Each item has its own activities that need to be performed and outputs that should be produced.

### 3.2. Practical Implementation and Outputs

In practice, the framework produces an analysis-ready dataset together with linked documentation outputs, most importantly the report accompanying the dataset. These outputs show how the dataset was defined, which preparation rules were applied, which records were changed or excluded, what data characteristics were identified during screening, and where the final documented version is stored. The purpose is that a researcher using the dataset can understand not only the final variables and records, but also how they were obtained.

Rule-based data preparation is organized through a cleaning catalogue and versioned rule sets. The cleaning catalogue contains predefined eligibility, validity, derivation, and transformation rules. For each dataset version, a rule set specifies which rules are active and which parameters are used, such as the age range that is eligible for the dataset. Rule execution produces rule execution logs, cleaning flags, and exclusion summaries, which allow users to see, for example, how many records were excluded by each eligibility rule and whether exclusions overlap. Those flags can later enable simple traceability of the records that were included, for instance, for a data submission for an international data call for a certain year, along with the reason for non-inclusion.

Screening is organized through screening catalogue items. Each item defines what aspect of the prepared dataset should be summarized, for which variables, using which output, and in which part of the report. Applicability fields allow screening items to be filtered and selected by dataset type, intended use, available variables, and analytical context. Within the assessment of missing and incomplete information, a value is considered missing if it is expected but absent, unknown if it is applicable but cannot be determined, and not applicable if the variable is structurally irrelevant for that record or dataset. Documentation should include a common core identifying the category of incomplete information, denominator, count, proportion, and assessment of variation across relevant structural variables. Additional items should be selected depending on the intended use and may include incomplete follow-up and cause-of-death ascertainment. The resulting screening outputs describe the study population, missingness, univariate distributions, multivariable distributions, and analysis-specific properties such as subgroup support for survival estimation.

When a dataset prepared through the framework is used in a scientific study, IDA findings may inform refinement of the statistical analysis plan, while the resulting analytical decisions belong to the subsequent analysis rather than to the IDA process itself. For example, assessment of the extent and structure of missing information may provide important guidance on the appropriateness of complete-case analysis, multiple imputation, or other missing-data approaches, but the selection and implementation of these methods form part of the statistical analysis plan [28]. In the resulting publication, the main Methods section should summarize the dataset definition, key eligibility and preparation rules, derivation of analysis variables, and IDA findings that affected the analysis or interpretation. The complete IDA report remains a technical document accompanying the dataset and may be referenced or provided as Supplementary Material where appropriate.

Together, these outputs demonstrate the practical value of the framework. The rule execution log shows which rules were applied or skipped, supporting reproducibility of the preparation process. The eligibility summary records the number of records excluded by each rule, making inclusion and exclusion decisions traceable. Screening outputs characterize the properties of the dataset, supporting analytical validity. Finally, dataset locking metadata link the final dataset version to the applied rule set, outputs, and IDA report, supporting reuse, auditability, and reconstruction of the dataset preparation process.

#### 3.2.1. Technical Prerequisites for Implementation

The implementation does not require the same technical infrastructure in all registries. Its generic structure is represented by the item set, which defines the activities and outputs required across the four stages. The content used to implement these items requires local customization according to the registry’s data structure, variable definitions, coding systems, validation procedures, intended uses, and governance arrangements. Broadly, there are two different ways of framework implementation. At the minimal level, only the basic technical requirements are needed, because the required outputs can be recorded in human-readable form in structured tables, reports, and procedural records. This level formalizes and connects practices that many registries may already perform in less standardized ways. At the more advanced level, machine-readable metadata can be integrated into computational workflows [4]. Rules can then be defined and executed centrally, and outputs generated directly from the data processing pipeline. The preparation logic remains the same on both levels, but the metadata-driven implementation can reduce manual effort, improve consistency, and facilitate comparison and reconstruction of the final dataset. In both cases, the essential requirement is that the procedural and final outputs, including the final report, are linked through a dataset version identifier to make clear what elements correspond to each other. Some parts of the process remain partly manual, particularly complex validity assessment and interpretation of screening findings. The framework does not remove the need for expert judgement, but makes this judgement visible and consistently documented.

#### 3.2.2. Relationship with Routine Registry Validation

Cancer registries already operate within established validation processes applied during data collection and maintenance, some of which are integrated into data entry and coding and therefore need to remain part of routine registry validation. In the proposed framework, they are documented as metadata about the prior validation context, while additional checks required for a specific dataset or analytical purpose are implemented as IDA cleaning rules. However, the IDA framework can be used to structure certain validation and quality-control activities, for a defined extracted dataset, as reflected in the optional screening item on internal registry monitoring. IDA may identify inconsistencies that were not resolved during routine validation. In such cases, the original value is not replaced, but rather the record is flagged, the applicable rule and finding are documented, and the action that should be taken, either manual review or retention with a warning, is recorded.

### 3.3. Demonstrative Use Case

#### 3.3.1. Dataset Preparation and Metadata Specification

The IDA framework was applied to lung cancer data extracted from the SCR to produce an illustrative survival dataset intended for the estimation of population-based survival among adult lung cancer patients diagnosed in Slovenia in 2011–2020. The initial extracted dataset contained 14,538 records, where the unit of observation is a new disease occurrence. Prior to data processing, the dataset was formally defined using structured metadata specifying its purpose, population, unit of analysis, time definition, and variable structure, which are presented in Supplementary File S2.

#### 3.3.2. Cleaning Results

Following the application of rules for validation, derivation and eligibility of records, the analysis-ready dataset was obtained. Eligibility rule application is summarized in Table 3. For each rule, the table reports both the number of records flagged by the rule and the number of records for which the rule was assigned as the primary exclusion reason. While some rules flag overlapping sets of records, the primary exclusion assignment enables transparent accounting of how many records are ultimately excluded by each rule. This allows us to assess the scope of each rule’s impact.

**Figure 1:**
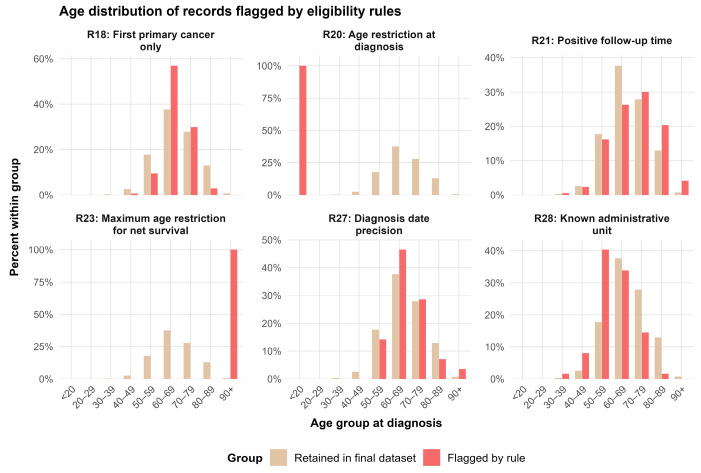
Age-group profile of records flagged by each eligibility rule. Each panel compares records flagged by the rule with records retained in the final dataset.

The characteristics of records affected by eligibility rules are summarized to assess how rule-based preparation shaped the final dataset. Figure 1 shows the age-group profile of records flagged by each eligibility rule and compares them to the retained records. This profile helps identify whether specific rules mainly affect particular groups, alerting the user to the potential change in the composition of the final data compared to the original dataset. In the example, rules R18 and R21 affect older groups more than younger ones, and rules R20 and R23 affect only the applicable group. A total of 565 records were excluded and the final analysis-ready dataset contained 13,973 records. A sensitivity analysis comparing observed survival in the pre-exclusion dataset and the final analysis dataset showed very similar estimates, with absolute differences below one percentage point at all evaluated time points.

#### 3.3.3. Screening Results

**Figure 2:**
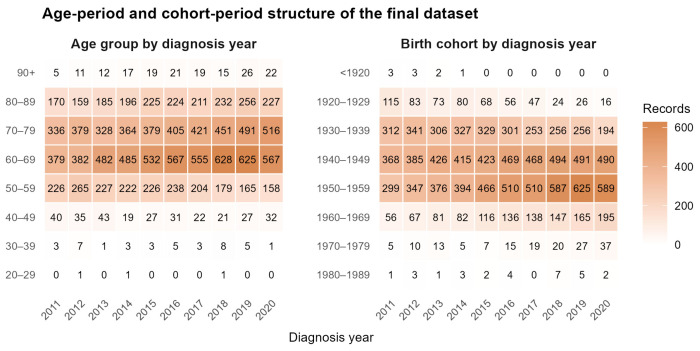
Age, diagnosis year, and birth cohort structure of the final dataset. Cells show the absolute number of records, providing a descriptive overview of how the composition of the dataset varies across diagnosis years and should be taken into consideration when making crude comparisons of outcomes over time. The heatmaps should be examined for concentrated and sparse cells which indicate which strata are well or poorly represented for further analysis. Unexpected gaps or discontinuities may indicate changes in coding or data preparation.

**Figure 3:**
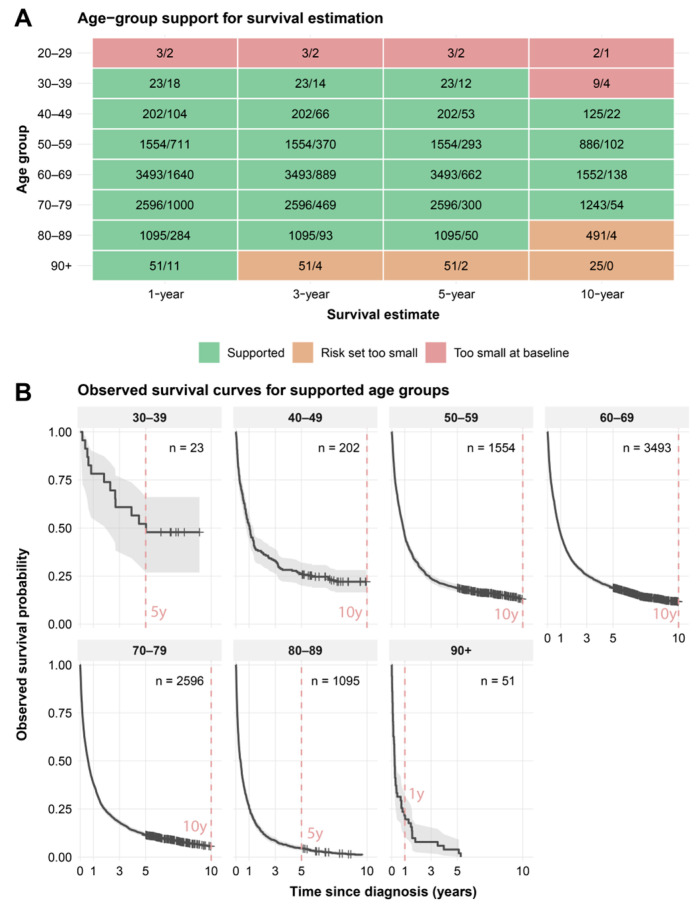
Subgroup-specific survival estimation feasibility. (**A**) Heatmap showing whether age subgroups among men supported estimation of 1-, 3-, 5-, and 10-year survival. For each time point, it is estimated that support is sufficient under two conditions: at least 10 cases diagnosed early enough to allow that length of follow-up and at least 10 cases still at risk at the time point. Cell labels show these two counts, respectively. (**B**) Observed survival curves for age subgroups that met the support criteria for at least one time point. Dashed vertical lines indicate the longest supported survival time for each subgroup.

**Figure 4:**
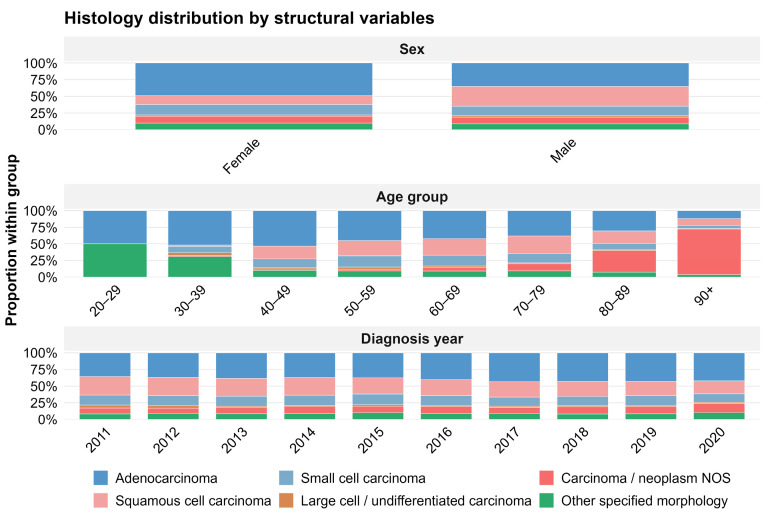
Histology distributions by structural variables of sex, 10-year age group, and diagnosis year.

Screening was performed to characterize the structure and analytical usability of the prepared dataset. This included evaluation of the final study population, assessment of missingness, univariate and multivariate distributions, and survival-specific assessments. As an example of population description screening, the age-period and cohort-period heatmaps (Figure 2) showed that records were concentrated mainly in the 60–69-year age groups and in birth cohorts from approximately 1950–1959, with increasing counts in later years. This illustrates how screening can reveal changes in the age and cohort composition of the final study population and show that crude comparisons across diagnosis years may partly reflect those differences rather than changes only in diagnosis, treatment or survival. The plots are descriptive and should not be interpreted as inferential evidence. Further, to assess analytical feasibility, subgroup-specific support for survival estimation was evaluated under predefined criteria based on the number of cases at baseline and the number remaining at risk over time. This enabled clear identification of subgroups with sufficient data to support reliable estimation of k-year survival (Figure 3). Support was strongest in middle- and older-adult age groups, while the youngest group (20–29 years) was not supported. These findings illustrate how screening can inform whether subgroup-specific estimates should be produced at all or interpreted with caution. Multivariable screening further examined the distribution of key registry variables across structural variables such as sex, age group, and diagnosis year. Figure 4 illustrates this for histology group. These summaries help identify whether clinically important categories are sufficiently represented across population subgroups and calendar periods, and whether sparse or unevenly distributed categories may affect planned stratified analyses. While histology distribution remains stable with diagnosis year, the distribution changes markedly with age groups.

#### 3.3.4. Structure of the Final Dataset and Outputs

The final analysis-ready dataset contained 16 variables, selected according to a pre-defined variable catalogue. In addition to the dataset itself, the framework generated a structured set of outputs, including rule definitions and rule set configurations, rule execution logs, eligibility exclusion summaries, screening summaries, and dataset locking metadata linking all outputs to a specific dataset version (Supplementary File S2).

## 4. Discussion

This article proposes a structured IDA framework for CRs. Its main contribution is creating a reproducible workflow to organize and formalize procedures in CRs by structuring the transitions from extracted registry data (data state B) to an analysis-ready dataset (C) while also retaining traceability to the operational registry database (A). While the proposed framework builds on the STRATOS IDA structure [4,5,6], it addresses the specific needs of CRs. As shown in the demonstration, preparation can be controlled through versioned rule sets and the resulting documentation explains the dataset in both a human-readable and machine-readable form. This is particularly relevant for external users of cancer registry data, since previous work has shown that data users may need clearer guidance on data preparation and interpretation after receiving registry data [29], and also responds to previous concerns that IDA activities are often insufficiently reported [30]. The framework places an emphasis on transparency and central use of metadata, extending its scope beyond existing CR data quality procedures. At the same time, the required activities can be implemented at different levels of technical capacity. The framework’s overall structure, represented by the item set, is expected to be transferable across registry settings. Local adaptation is nevertheless required because registries differ in their source data structures, data collection practices, coding systems, validation procedures, intended uses, governance, and legal requirements. These differences may affect which rules are applicable, which screening items are informative, how dataset versions are documented, and which outputs can be shared with data users. Registries with limited technical resources may initially rely on human-readable documentation and partly manual execution, whereas those with established metadata systems and computational pipelines may implement a more automated approach. Additional challenges may arise where data are collected from heterogeneous sources, follow-up procedures are incomplete, historical coding changes are poorly documented, or legal restrictions limit linkage, access, or dissemination of detailed outputs. The framework therefore provides a common structure, but its implementation must remain proportionate to the registry’s purpose, resources, data environment, and regulatory context. Although developed in a population-based cancer registry, the framework’s general structure is also applicable to institutional, disease- or intervention-specific, or direct-to-patient registries, provided that the locally defined rules and screening items reflect the registry’s purpose, population coverage, data sources, and follow-up procedures.

In this article, we focus on methodological development and demonstration, rather than evaluating performance. A limitation of the present study is that it does not include a formal comparative evaluation against conventional unstructured data preparation workflows. Therefore, although the proposed framework is designed to support traceability, reproducibility, and more complete documentation, the present demonstration should not be interpreted as empirical evidence that it reduces errors, saves time, or improves inter-analyst agreement. Such outcomes require dedicated comparative studies, for example by assigning the same registry dataset to multiple analysts using structured and unstructured workflows and comparing the resulting datasets, processing decisions, completeness of documentation, and time requirements. The demonstrative use case nevertheless shows that the framework can be implemented in practice and that adapting the general IDA framework to a CR setting is feasible and necessary. Further applications are needed to develop the proposed item set into a validated checklist that will support alternative uses (e.g., incidence reporting, international submissions, or internal validation) within the same framework.

We have identified several directions for future work. First, systematic application and validation for additional registry tasks and datasets from different time periods is needed, including comparative evaluations of structured and conventional data preparation workflows. Further development and standardization of computational tools that support metadata-driven execution of the full IDA process are required, with the intention to be transferred to other registries that adopt the metadata schema. Another direction is further technological integration with metadata catalogues, ontologies, and validation environments, which would strengthen interoperability and reduce manual maintenance of rules [17]. Application in other cancer registries will be necessary to assess which parts of the framework are broadly transferable and which require local adaptation. The structured metadata approach could also support future alignment with emerging health data metadata standards, such as HealthDCAT-AP within the European Health Data Space [31,32]. This is consistent with real-world data quality frameworks that emphasize data quality across the data life cycle, not only at final analysis [33]. The framework also improves provenance, interpretability, and reusability of registry data for secondary analyses, which are central concerns of the FAIR principles [34].

## 5. Conclusions

The proposed framework addresses an important gap between general IDA method- ology and real-world CR data, providing a transparent and reproducible approach to preparing analysis-ready datasets for various uses of CR data. It supports the need for adapting existing IDA frameworks by identifying registry-specific requirements. The lung cancer survival use case demonstrates how these requirements can be addressed in a structured workflow.

## Figures and Tables

**Table 1:** Comparison of the STRATOS IDA framework and the proposed cancer-registry-specific adaptations.

Aspect of STRATOS IDA Requiring Registry-Specific Operationalization	Operationalization in the Present Cancer Registry IDA Framework
Primarily formulated for single-study datasets with a defined research plan and analysis strategy	Designed for continuously updated cancer registries with repeated data extraction and multiple analytical uses
Dataset identity is usually assumed to be defined by the study design	Dataset identity is formalized through three states: operational registry data, extracted dataset, and analysis-ready dataset
Metadata describe data context but do not control registry dataset generation	Metadata define dataset scope, analytical purpose, population, unit, variables, coding systems, rule context, rule sets, and versioning
Rule versions and classification versions are not required as explicit operational elements	Versioned rule sets, coding system context, validation tool context, and dataset identifiers are recorded
Cleaning is defined generally as error detection and correction	Cleaning is operationalized through predefined validity, eligibility, derivation, and transformation rules, with execution logs, flags, and exclusion summaries
Screening is not tailored to registry outputs	Screening catalogues include registry- and use-specific outputs, such as cohort derivation, missing/unknown values, follow-up support, and survival-specific subgroup support
Reporting communicates IDA findings but does not define dataset locking	Reporting includes dataset locking, output linkage, retrievability of outputs, version metadata, and an IDA report accompanying the dataset
Reproducibility is a general principle	Reproducibility is operationalized through linked metadata, rule logs, versioned outputs, file paths, and file integrity identifiers

**Table 2:** Summary of the structured IDA framework for CRs.

Stage	Purpose	Summary of Included Items	Main Outputs
Metadata	Define the dataset and the context in which it will be prepared	Define the IDA scope and intended use; population and unit of analysis, source data and extraction context; variables and coding systems; rule context and rule set; dataset and version identifiers	Definitional metadata, variable catalogue, rule definitions, rule-set specification
Cleaning	Apply predefined rules to produce the analysis-ready dataset	Execute validity rules, derive or transform variables, apply eligibility criteria	Analysis-ready dataset, rule execution log, cleaning flags, exclusion summary
Screening	Characterize the prepared data in relation to its intended use	Describe the final study population; assess missing and incomplete information; examine univariate and multivariable distributions, and analysis-specific properties	Screening tables, figures, summaries, documented findings and limitations
Reporting	Lock the final dataset and connect it with its documentation	Define the final dataset version; link it to the source data, extraction context, applied rule set, cleaning outputs, and screening results; compile the IDA report	IDA report, locking and version metadata

**Table 3:** Eligibility rule application in the demonstrative dataset.

Rule ID	Rule Name	Number of Records Flagged by Rule	Number of Records Excluded by Rule
R18	First primary cancer only	137	137
R20	Age restriction at diagnosis	4	4
R21	Follow-up time above zero days	339	335
R22	Completeness for net survival	0	0
R23	Maximum age restriction for net survival	15	14
R27	Diagnosis date precision	28	27
R28	Known administrative unit	62	48

## Data Availability

No new data were created in this study. The individual-level registry data used in the demonstrative use case are not publicly available due to legal and privacy restrictions. The R code, metadata templates, simulated demonstration data, and example outputs supporting the implementation are available in Zenodo at https://doi.org/10.5281/zenodo.21289195 (accessed on 10 July 2026) and the development repository, version 1.0.0, is available on GitHub at https://github.com/mjurtela/cr-ida-demo (accessed on 10 July 2026).

## Supplementary Materials

The following supporting information can be downloaded at: https://www.mdpi.com/article/10.3390/cancers18142332/s1, Supplementary File S1: Item set of the structured initial data analysis framework for cancer registries; Supplementary File S2: Demonstrative IDA report for a survival dataset.

## Abbreviations

The following abbreviations are used in this manuscript:

CR	Cancer registry
ECIS	European Cancer Information System
ENCR	European Network of Cancer Registries
IACR	International Association of Cancer Registries
IDA	Initial data analysis
SCR	Slovenian Cancer Registry
