# Supplementary material for: Initial Data Analysis for Cancer Registries: A Structured Framework and Demonstration Using Slovenian Cancer Registry Data
Source: Cancers (Basel). 2026 Jul 20;18(14):2332. doi: 10.3390/cancers18142332 (PMC13407080; doi:10.3390/cancers18142332)
Supplement: Supplementary file 1 [file cancers-18-02332-s001.zip › Supplementary File S1.pdf]

Supplementary File S1: Item set of the structured initial data analysis framework for cancer registries

Table 1: Items of the structured initial data analysis framework for cancer registries, organised by metadata, cleaning, screening, and reporting.

**1 Metadata**				
**Item no**	**Topic**	**Specification**	**Required outputs**	**Example of relevance**
1.1	IDA scope for the dataset defined	The dataset must be clearly defined as a specific, fixed extract from the database (data state A) with an explicit start (extraction moment – data state B) and end (final, locked version – data state C).	Extraction moment; definition of final dataset	If two analysts use the same dataset but extracted at different times, then the analysis results may differ.
1.2	Intended use and analytical purpose	The purpose of the dataset must be declared before preparation begins, as it determines all downstream decisions including variable selection, eligibility, and cleaning rules.	Declared purpose	If a dataset prepared for incidence is reused for survival analysis without adjustment, the results may differ, and also be unreproducible.
1.3	Population	The dataset must define which population it represents, including disease definition, inclusion criteria and any restrictions relevant to its use.	Population definition with inclusion/exclusion criteria	Including all cancer cases can be interpreted differently from restricting cases to the first primary tumour only.
1.4	Unit of analysis	The unit represented by each row must be clearly defined (e.g., tumour, person, event).	Defined unit of analysis	Unmonitored mixing of tumour- and patient-level data may lead to overestimation of cases.
1.5	Time definition	The time scope and meaning of time variables must be defined, including diagnosis period, follow-up period, or censoring reference.	Time window; follow-up definition if relevant	Results may differ for different follow-up periods.
1.6	Data sources and extraction logic	The source of the data and their linkage as well as the rules used to extract it must be described in a way that allows reconstruction of the dataset.	Sources of the data; extraction rules	Undocumented filtering in code prevents reproducibility.

Table 1: Items of the structured initial data analysis framework for cancer registries, organised by metadata, cleaning, screening, and reporting. Continued.

**Item no**	**Topic**	**Specification**	**Required outputs**	**Example of relevance**
1.7	Validation context and prior data validation	The dataset must document which validation tools, versions, and internal quality control procedures were applied during data collection and prior to extraction.<br>This is needed because IDA should not duplicate registries' validation systems already in place, but must make them visible and traceable.<br>Where multiple validation layers are used, their relationship and order of application must be clearly defined. If additional validation checks are performed during IDA, they must be specified as part of the cleaning rule set (item 1.9).	Validator tools and versions (e.g., ENCR validator v2.2.8, IARC tool release, internal script-based validation); description of internal validation procedures; if applicable, reference to validation rules applied within IDA	Dataset prepared in 2023 using validator v2.1 and in 2026 using v2.2, but validator version is not recorded.<br>Differences in validation rules may lead to different error flags and exclusions. As a consequence, datasets are not comparable and differences cannot be explained.
1.8	Variable definitions	The dataset must define which variables are included and how key variables should be interpreted.<br>Define how missing, unknown, and not applicable values are represented and distinguished across the dataset.<br>Specify whether missingness is structural (by design), unknown, or not applicable.	Variable list with key variable descriptions; defined missing-value conventions	Stage variable without specifying staging system and edition.<br>Event variable without specifying what it represents.<br>Using the same codes for unknown and not applicable.
1.9	Coding systems and rule context	Define all classification systems and coding schemes used for variables (e.g., disease classification, staging systems, morphology coding), including their versions and, where applicable, periods of use.<br>Define the complete rule framework applied during IDA, including eligibility rules, cleaning rules, and derivation rules, prior to execution.	List of coding systems with versions and validity periods, if applicable; rule set identifier (versioned); eligibility rules; cleaning rules; derivation rules	Use of multiple classification systems without specifying versions (e.g., ICD-O editions or TNM editions applied across different years).<br>Rules applied during data preparation without a defined rule set or without documentation of eligibility, cleaning, or derivation logic.

Table 1: Items of the structured initial data analysis framework for cancer registries, organised by metadata, cleaning, screening, and reporting. Continued.

Item no	Topic	Specification	Required outputs	Example of relevance
1.10	Dataset versioning scheme	The versioning scheme must be defined specifying how each dataset version will be uniquely identified. This includes defining how dataset identifiers are generated and how different versions relate to each other.	Defined dataset ID generation approach with rules for version increments; defined linkage between dataset and associated outputs	Multiple files with no clear distinction create uncertainty about which dataset was used for analysis and prevent reproducibility of results.<br>Dataset ID is generated using dataset context, timestamp, and rule set version, with associated logs stored in a defined location, so each dataset can be uniquely identified.<br>A hash of the data file (e.g., SHA-256) is computed to uniquely identify the locked dataset and remains unchanged if the file remains unchanged.
1.11	Metadata storage	The location and structure of metadata and associated outputs, such as logs and reports, must also be defined to ensure that all elements of the dataset state can be retrieved and linked.	Defined storage location for dataset, metadata, and reports	If dataset, rule execution log, cleaning summaries, screening outputs, and the IDA report are stored in separate folders without a shared dataset identifier or directory structure, they cannot be reliably linked to the exact dataset version.
**2 Cleaning**				
**Item**	**Topic**	**Specification**	**Required outputs**	**Example of relevance**
2.1	Apply validity rules	Apply all predefined validity rules, including structural, format, and logical consistency checks, according to the defined rule set.	Dataset with applied cleaning and validity flags;<br>List of applied rules (rule execution log);<br>Flagged records with issues	Date of death recorded before date of diagnosis; inconsistent variable coding across records; invalid or out-of-range values detected during rule-based checks.
2.2	Apply corrections and flag unresolved issues	Apply corrections only where rules explicitly permit them and record all changes. Where correction is not possible, flag or handle records according to policy.	Correction log;<br>List of flags for unresolved inconsistencies;<br>List of handled records	Manually adjusted incorrect values without documentation of corrections may hinder reproducibility of steps and results.
2.3	Apply and document eligibility decisions	Apply eligibility criteria defined in metadata and record inclusion and exclusion decisions explicitly. Keep eligibility separate from validity.	Inclusion and exclusion flags with reasons for exclusion	Records excluded informally during analysis.

Table 1: Items of the structured initial data analysis framework for cancer registries, organised by metadata, cleaning, screening, and reporting. Continued.

Item no	Topic	Specification	Required outputs	Example of relevance
2.4	Generate derived variables	Create required derived variables using predefined definitions and document the derivation logic.	Derived variables and documented derivation rules	Follow-up time is calculated differently between analyses.
2.5	Preserve data versions and ensure reproducibility	Document all cleaning steps and maintain linkage between original and cleaned datasets.<br>Confirm that applying the same rules produces the same result and that the dataset is fully valid.	Original dataset;<br>Cleaned dataset with version linkage;<br>Reproducibly cleaned dataset	The dataset is modified without a record of changes.<br>Re-running cleaning produces different outputs due to undocumented manual steps.
2.6	Optional: Transform and harmonise data	Apply predefined transformations required to prepare the dataset structure for the intended use by harmonizing variable formats and coding systems, linking or merging external data sources, and creating analysis-specific dataset layouts.	Transformed analysis dataset;<br>Documented transformation and harmonization rules;<br>Linkage or merging log if applicable	External mortality data are linked to registry records to derive vital status and follow-up time; tumour-level records are restructured to patient-level data.
**3 Screening**				
**Item no**	**Topic**	**Specification**	**Required outputs**	**Example of relevance**
3.1	Define screening scope based on analytical purpose	Identify the key variables, structural variables, subgroups, and dataset-specific dimensions to be assessed based on the intended use of the dataset.	Defined screening scope; list of key variables; list of structural variables; list of dataset-specific screening items	If screening outputs are produced without predefined scope, important findings may be overlooked among irrelevant summaries, or too many outputs are produced with little focus.
3.2	Describe cohort or study population definition	Characterize the prepared dataset in terms of the final cohort or study population, unit of analysis, inclusion period.	Cohort/study population description; final dataset size; unit of analysis	If the final study population is not clearly described, later screening findings cannot be interpreted in relation to the actual dataset.
3.3	Assess missing data, unspecified values, and completeness indicators	Evaluate the extent and structure of missing, unknown, unspecified, not applicable, or otherwise incomplete information across key variables and relevant subgroups.	Missingness summaries; Unspecified value summaries; completeness indicators	Missing or unknown stage concentrated in older cases may affect interpretation of stage distribution and subgroup analyses.

Table 1: Items of the structured initial data analysis framework for cancer registries, organised by metadata, cleaning, screening, and reporting. Continued.

Item no	Topic	Specification	Required outputs	Example of relevance
3.4	Examine univariate descriptions	Review distributions and summary statistics of important variables one at a time to understand the structure of the prepared dataset.	Frequency tables; summary statistics; distribution summaries	Unexpected patterns may indicate coding, extraction, or transformation issues.
3.5	Examine multivariable descriptions	Examine relationships between variables to identify subgroup patterns relevant for interpretation.	Cross-tabulations; Stratified summaries by structural variables	Stage distribution may differ strongly by age or sex; histology may be sparse in some subgroups.
3.6	Perform dataset-specific screening	Apply additional screening items required by the dataset type, analytical purpose, or planned analysis.	Dataset-specific screening summaries; feasibility checks; analysis-support summaries	In a survival dataset, this may include follow-up support, number at risk, risk-set depletion, loss to follow-up, and subgroup-specific support for estimating k-year survival.
3.7	Optional: perform registry process monitoring for internal use	When IDA is used for internal registry purposes, assess indicators related to registry processes, coding practices, validation or linkage processes, for example.	Internal registry-process indicators	Validation flag rates, linking results, delayed registration, shifts after coding-rule changes may be monitored internally.
**4 Reporting**				
**Item no**	**Topic**	**Specification**	**Required outputs**	**Example of relevance**
4.1	Define the final dataset	The dataset must be explicitly defined as final and analysis-ready.<br>Assign a clear, unique version ID to the dataset and use it consistently in all outputs, analyses, and documentation.	Dataset version ID;<br>Defined naming approach defined	Dataset used without a clear final version.<br>If multiple final files exist, there is uncertainty about which dataset produced the results.
4.2	Link the dataset to extraction and source data	Record when and and under which conditions the dataset was extracted to ensure traceability to the source data.	Extraction date, time;<br>Extraction query;<br>Reference to source	If the dataset cannot be linked to a specific extraction, the data cannot be reconstructed.
4.3	Link dataset to rule framework	The dataset must be explicitly linked to the rule set used for its preparation, including rule set version.	Rule set identifiers (rule versions linked to dataset version)	If results differ but rule changes are recorded, differences can be transparently explained without needing to investigate the code.

Table 1: Items of the structured initial data analysis framework for cancer registries, organised by metadata, cleaning, screening, and reporting. Continued.

Item no	Topic	Specification	Required outputs	Example of relevance
4.4	Compile the IDA report	All outputs generated during cleaning and screening must be compiled and structured for interpretation.	Cleaning summaries; screening outputs; structured metadata	Cleaning and screening outputs exist but are scattered across files or scripts, making interpretation difficult.
4.5	Ensure linking and retrievability	Dataset, its metadata and the IDA report are linked through consistent identifiers and stored in a location where they can be retrieved.	IDA report;<br>Linked dataset and metadata;<br>References connecting them	Files stored separately without linkage means information cannot be connected or verified.
4.6	Provide interpretation context	Key dataset characteristics, limitations, and constraints relevant to the intended analysis need to be presented.	Interpretation text	Results are interpreted without context on limitations such as missing data, follow-up constraints, or subgroup sparsity.
4.7	Preserve reproducibility and traceability	All components required to reproduce the dataset must be available and versioned. Any change must produce a new version.	Stored rules, logs, inputs, outputs	Dataset is modified or regenerated without version tracking, making results impossible to reproduce.
