# Supplementary material for: Initial Data Analysis for Cancer Registries: A Structured Framework and Demonstration Using Slovenian Cancer Registry Data
Source: Cancers (Basel). 2026 Jul 20;18(14):2332. doi: 10.3390/cancers18142332 (PMC13407080; doi:10.3390/cancers18142332)
Supplement: Supplementary file 1 [file cancers-18-02332-s001.zip › Supplementary File S2.pdf]

Supplementary File S2: Demonstrative IDA report for a survival dataset

Initial Data Analysis Report

Survival dataset for external researchers

Slovenian Cancer Registry

Date of IDA initialization: 2026-07-10

Contents

1 Purpose of this report **1**

2 Dataset overview **1**

2.1 Population and unit of analysis . . . 1

2.2 Data source and extraction logic . . . 1

2.3 Prior validation context and rule context . . . 2

3 Cleaning results **3**

3.1 Rule execution log . . . 3

3.2 Cleaning flag summary . . . 3

3.3 Eligibility exclusion summary . . . 4

3.4 Properties of retained and excluded records . . . 4

3.5 Rule-specific profiles of flagged records . . . 5

3.6 Sensitivity analysis of eligibility exclusions . . . 8

4 Screening results **8**

4.1 Cohort definition . . . 8

4.2 Missingness and incomplete information . . . 12

4.3 Univariate distributions . . . 15

4.4 Multivariate screening . . . 21

4.5 Survival-specific aspects . . . 23

5 Final analysis-ready dataset **27**

5.1 Final dataset dimensions . . . 27

5.2 Variables included in the final dataset . . . 28

6 Reproducibility and locking **29**
6.1 Dataset status 29
6.2 Locking metadata recorded for this dataset 29
6.3 Links to outputs related to the final dataset 29

7 Session information **29**

1 Purpose of this report

This report documents the initial data analysis (IDA) process for dataset **LUNG_SURV_2020_v2**, prepared for **overall survival analysis**. It summarizes the dataset definition, preparation context, selected metadata, screening summaries, and the final variable catalogue. It is intended for external researchers to receive along with the requested dataset and to be examined in order to understand how data was produced and potential data properties and limitations that may affect the intended statistical analyses.

2 Dataset overview

Table 1: Dataset overview

Element	Definition
Dataset ID	LUNG_SURV_2020_v2
Short description	Demonstrative lung cancer survival dataset prepared from the Slovenian Cancer Registry
Analytical purpose	Overall survival estimation
Cancer site	Lung cancer, ICD-10 C33–C34
Case-index	Incident lung cancer cases diagnosed between 2011 and 2020; first primary tumour per patient
Diagnosis period	2011–2020
Follow-up end	31 December 2025
Spatial coverage	Slovenia
Language	English
Unit of analysis	Disease/tumour-level record; one row represents one eligible lung cancer diagnosis
Main exclusion criteria	Diagnoses outside the study period; non-first primary tumours; age outside eligible range; zero or invalid follow-up time if excluded by the active rule set; additional rule-set-specific exclusions
Extraction date	1 April 2026

2.1 Population and unit of analysis

The population was defined as **Adult lung cancer patients (SCR, 2011–2020)**, with inclusion based on **First primary lung cancer (ICD-10 C33–C34), diagnosed 2011–2020, recorded in the Slovenian Cancer Registry**. The unit of analysis was **tumour**: one tumour diagnosis per patient (first primary). Records were linked to patient-level information through **linked to patient via registration_number**.

2.2 Data source and extraction logic

The dataset was extracted from the Slovenian Cancer Registry operational database. The extraction was performed defined by the extraction date recorded in the dataset metadata: **2026-03-15**. The extraction selected lung cancer records using ICD-10 codes **C33–C34** and diagnosis years **2011–2020**. The source extraction was performed through a scripted SQL query linking registry resident, disease, demographic, vital status, morphology, stage, diagnosis basis, finding, administrative area, and institutional information. The SQL extraction code is archived as part of the reproducible framework code for this dataset. The extracted dataset therefore represents a time-specific registry extract prepared for the declared analytical purpose. The extracted dataset contained **14,538** records.

2.3 Prior validation context and rule context

Cancer registry data undergo routine validation and quality-control procedures before extraction. These procedures are part of the interpretation context of the extracted dataset and are reported separately from the IDA rules applied during preparation. Prior validation context describes checks and classification systems already relevant to the registry data before IDA. IDA-specific validity, eligibility, and derivation rules are then documented in the rule execution log in the cleaning section.

Table 2: Prior validation context, classification systems, and rule context.

version	valid_from	valid_to	notes
ICD-O-3	2001-01-01	2011-12-31	NA
ICD-O-3 v1	2012-01-01	2019-12-31	NA
ICD-O-3 v2	2020-01-01	2300-12-31	NA
TNM, 6th edition	2010-01-01	2016-12-31	applied for lung (approx. from 2009/2010)
TNM, 7th edition	2017-01-01	2300-12-31	NA
ENCR data validation tool v2.2.8	2018-01-01	2300-12-31	replaced IARC tool
IARC data validation tool	2010-01-01	2018-12-31	NA

The table above is intended to make visible which classification systems, validation tools, and internal registry procedures form the context for the extracted data. It should not be interpreted as a replacement for the rule execution log. The rule execution log below records which IDA preparation rules were actually applied to this dataset version.

3 Cleaning results

This section summarizes the rule-based preparation of the dataset, including applied rules, cleaning flags, eligibility exclusions, and descriptive summaries of records retained and excluded during preparation. The retained/excluded summaries are based on structural variables and are used to document how eligibility rules affected the composition of the final analysis-ready dataset.

3.1 Rule execution log

The rule execution log records which rules were applied or skipped during preparation. It provides the link between the predefined rule set and the actual processing of the dataset.

Table 3: Applied and skipped rules during dataset preparation.

Rule ID	Rule name	Executed	Records affected	Comment
R01	Date standardization	yes	0	diag_date, death_date and birth_date converted to Date.
R02	Vital status standardization	yes	22	Mapped vital_status_code 9 -> dead, 1 -> alive; all others set to NA.
R03	Diagnosis date imputation	no	0	Skipped: no partial diagnosis dates available in current extract.
R04	Coding system harmonization	no	0	Skipped for current demo: context-dependent classification harmonization not yet implemented.
R05	Duplicate record detection	yes	0	Flagged records duplicated on registration_number + diag_date + disease_id.
R06	Temporal consistency of dates	yes	77	Flagged records with impossible date order.
R08	Extreme survival time check	yes	0	Flagged surv_time_days < 0 or > 16 years.
R09	Administrative follow-up end	yes_already_derived	14538	followup_end_date already set to 2025-12-31
R10	Censor beyond follow-up	yes	1829	Deaths after administrative follow-up end censored.
R11	Lost-to-follow-up handling	yes_flag_only	22	Flagged records with vital_status_code == 0; death_date represents last known system date.
R12	Event indicator	yes_already_derived	22	Event already derived earlier from vital_status_clean, death_date and followup_end_date.
R13	Survival time calculation	yes_already_derived	22	surv_time_days and surv_time_years already derived earlier from end_date - diag_date.
R14	Sex recoding for net survival	yes	0	Recode for ratetable linkage: 1 -> male, 2 -> female, else NA.
R17	Expected mortality linkage eligibility	yes	15	Flagged records not eligible for expected mortality linkage.
R18	First primary cancer only	yes	137	Implemented as earliest diag_date within registration_number; ties resolved by smallest disease_id.
R20	Age restriction at diagnosis	yes	4	Applied from rule_parameters: age_min = 20, age_max = 120.
R21	Positive follow-up time	yes	339	Zero follow-up excluded.
R22	Completeness for net survival	yes	0	Required variables for life-table linkage checked.
R23	Maximum age restriction for net survival	yes	15	Applied from rule_parameters: age_max_net = 95.
R24	Valid sex for net survival	not_active	0	Not required for this rule set.
R25	Valid diagnosis basis	not_active	0	Not required for this rule set.
R26	Known stage restriction	not_active	0	Known stage not required for this rule set.
R27	Diagnosis date precision restriction	yes	28	Excluded records where only diagnosis year is valid; month-year and complete dates retained.
R28	Known administrative unit restriction	yes	62	Excluded records with administrative unit NEZNANO.

3.2 Cleaning flag summary

Cleaning flags summarize records affected by validity checks, derivations, transformation, and other preparation steps. These counts describe issues detected or handled during rule-based preparation.

dataset_id	rule_set_id	n_records_input	n_unknown_vital_status	n_duplicate_flag	n_date_order_problems
LUNG_SURV_2020_v2	RS_SURV_LUNG_DEMO_v2	14538	22	0	77

3.3 Eligibility exclusion summary

Eligibility rules define which records are retained in the final analysis-ready dataset. The summary below reports the number of records failing each eligibility rule and the number for which the rule was recorded as the primary exclusion reason.

Table 5: Eligibility exclusion summary.

Rule set ID	Rule ID	Rule name	Records failing rule	Primary exclusion reason
RS_SURV_LUNG_DEMO_v2	R18	First primary cancer only	137	137
RS_SURV_LUNG_DEMO_v2	R20	Age restriction at diagnosis	4	4
RS_SURV_LUNG_DEMO_v2	R21	Positive follow-up time	339	335
RS_SURV_LUNG_DEMO_v2	R22	Completeness for net survival	0	0
RS_SURV_LUNG_DEMO_v2	R23	Maximum age restriction for net survival	15	14
RS_SURV_LUNG_DEMO_v2	R27	Diagnosis date precision	28	27
RS_SURV_LUNG_DEMO_v2	R28	Known administrative unit	62	48

3.4 Properties of retained and excluded records

The following tables compare records retained in the final analysis-ready dataset with records excluded during preparation. The comparison is shown for structural variables only: diagnosis year, age group, and sex. Because the proportion excluded is small in most groups, the information is presented in tables rather than as retained/excluded proportion plots.

3.4.1 Retained and excluded records by diagnosis year

Table 6: Records retained and excluded by diagnosis year.

Diagnosis year	Record status	Records	Total in group	Percent within group
2011	Retained in final dataset	1,159	1,216	95.3%
2011	Excluded before final dataset	57	1,216	4.7%
2012	Retained in final dataset	1,239	1,286	96.3%
2012	Excluded before final dataset	47	1,286	3.7%
2013	Retained in final dataset	1,278	1,322	96.7%
2013	Excluded before final dataset	44	1,322	3.3%
2014	Retained in final dataset	1,307	1,338	97.7%
2014	Excluded before final dataset	31	1,338	2.3%
2015	Retained in final dataset	1,411	1,477	95.5%
2015	Excluded before final dataset	66	1,477	4.5%
2016	Retained in final dataset	1,491	1,554	95.9%
2016	Excluded before final dataset	63	1,554	4.1%
2017	Retained in final dataset	1,435	1,493	96.1%
2017	Excluded before final dataset	58	1,493	3.9%
2018	Retained in final dataset	1,535	1,606	95.6%
2018	Excluded before final dataset	71	1,606	4.4%
2019	Retained in final dataset	1,595	1,659	96.1%
2019	Excluded before final dataset	64	1,659	3.9%
2020	Retained in final dataset	1,523	1,587	96%
2020	Excluded before final dataset	64	1,587	4%

3.4.2 Retained and excluded records by age group

Table 7: Records retained and excluded by age group at diagnosis.

Age group	Record status	Records	Total in group	Percent within group
<20	Retained in final dataset	0	4	0%
<20	Excluded before final dataset	4	4	100%
20-29	Retained in final dataset	6	6	100%
20-29	Excluded before final dataset	0	6	0%
30-39	Retained in final dataset	52	54	96.3%
30-39	Excluded before final dataset	2	54	3.7%
40-49	Retained in final dataset	360	370	97.3%
40-49	Excluded before final dataset	10	370	2.7%
50-59	Retained in final dataset	2,478	2,570	96.4%
50-59	Excluded before final dataset	92	2,570	3.6%
60-69	Retained in final dataset	5,259	5,456	96.4%
60-69	Excluded before final dataset	197	5,456	3.6%
70-79	Retained in final dataset	3,898	4,055	96.1%
70-79	Excluded before final dataset	157	4,055	3.9%
80-89	Retained in final dataset	1,811	1,885	96.1%
80-89	Excluded before final dataset	74	1,885	3.9%
90+	Retained in final dataset	109	138	79%
90+	Excluded before final dataset	29	138	21%

3.4.3 Retained and excluded records by sex

Table 8: Records retained and excluded by sex.

Sex	Record status	Records	Total in group	Percent within group
Female	Retained in final dataset	4,956	5,137	96.5%
Female	Excluded before final dataset	181	5,137	3.5%
Male	Retained in final dataset	9,017	9,401	95.9%
Male	Excluded before final dataset	384	9,401	4.1%

3.5 Rule-specific profiles of flagged records

The following figures describe the structural profile of records flagged by each eligibility rule. These profiles are based on the independent rule flags, not on the primary exclusion reason. This is important because a record can fail more than one eligibility rule, while the primary exclusion reason depends on the order in which rules are recorded. Therefore, the figures should be interpreted as profiles of records flagged by each rule, compared with records retained in the final dataset.

Rule-specific profiles are shown by age group, diagnosis year, and sex in Figures 1, 2, and 3.

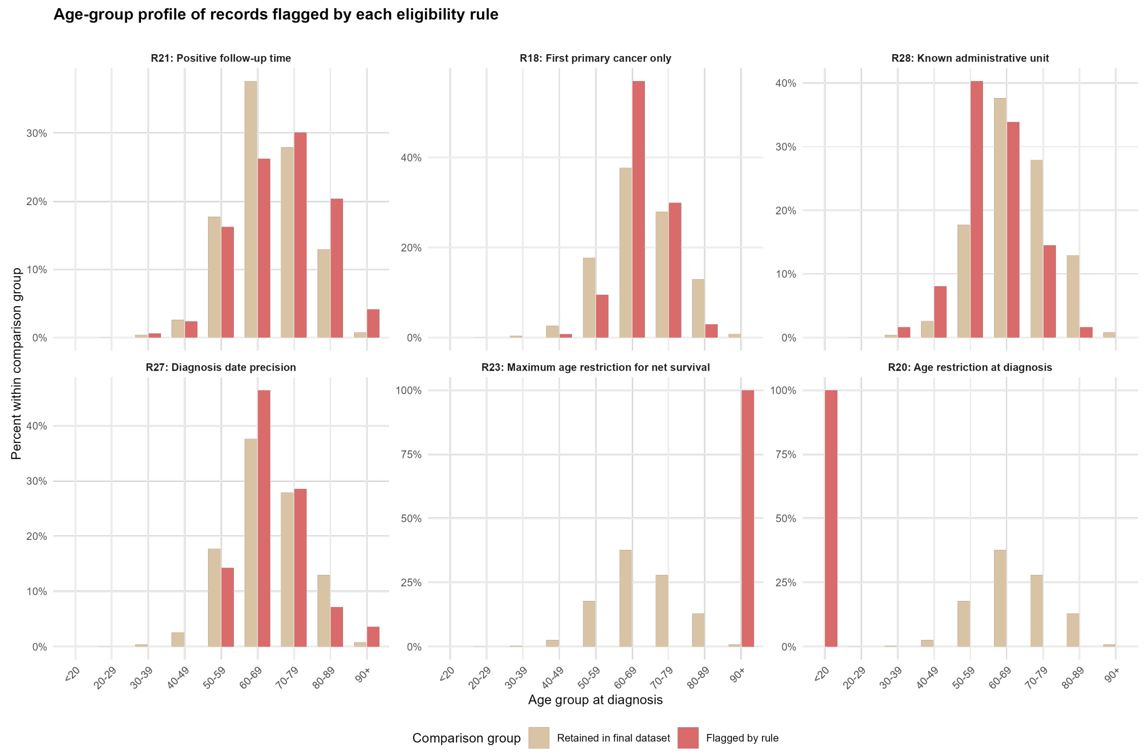

Figure 1: Age-group profile of records flagged by each eligibility rule, compared with records retained in the final dataset.

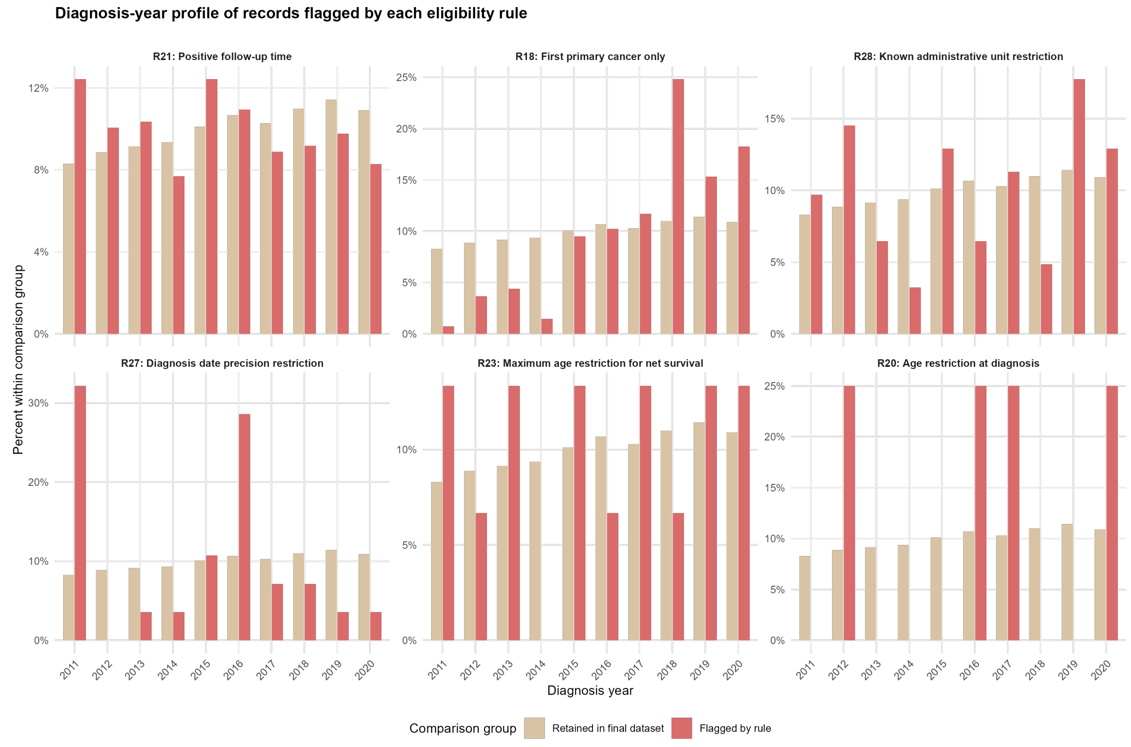

Figure 2: Diagnosis-year profile of records flagged by each eligibility rule, compared with records retained in the final dataset.

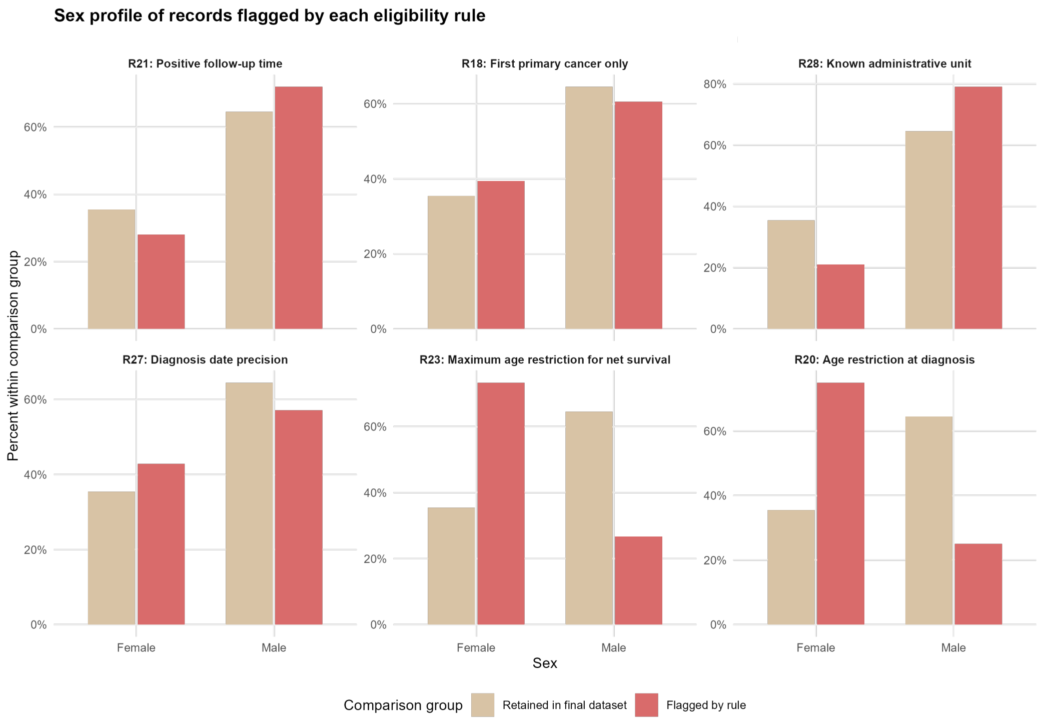

Figure 3: Sex profile of records flagged by each eligibility rule, compared with records retained in the final dataset.

3.6 Sensitivity analysis of eligibility exclusions

To assess whether application of the eligibility criteria materially affected overall survival estimates, observed survival was compared between the pre-exclusion dataset and the final analysis dataset. Observed survival was slightly higher in the final dataset at all evaluated time points, with absolute differences below one percentage point (Table 9; Figure 4). The comparison therefore indicates only a small effect of exclusions on overall observed survival. However, it does not exclude the possibility of larger effects within specific subgroups.

Table 9: Kaplan–Meier observed survival estimates before and after application of eligibility criteria.

Dataset	Time since diagnosis, years	Number at risk	Number of events	Observed survival, %	Standard error, %	Lower 95% CI, %	Upper 95% CI, %
Pre-exclusion dataset	1	6480	8036	44.64	0.41	43.83	45.45
Pre-exclusion dataset	3	3516	2964	24.22	0.36	23.53	24.92
Pre-exclusion dataset	5	2609	907	17.97	0.32	17.35	18.60
Final analysis dataset	1	6345	7628	45.41	0.42	44.58	46.23
Final analysis dataset	3	3431	2914	24.55	0.36	23.84	25.27
Final analysis dataset	5	2549	882	18.24	0.33	17.61	18.89

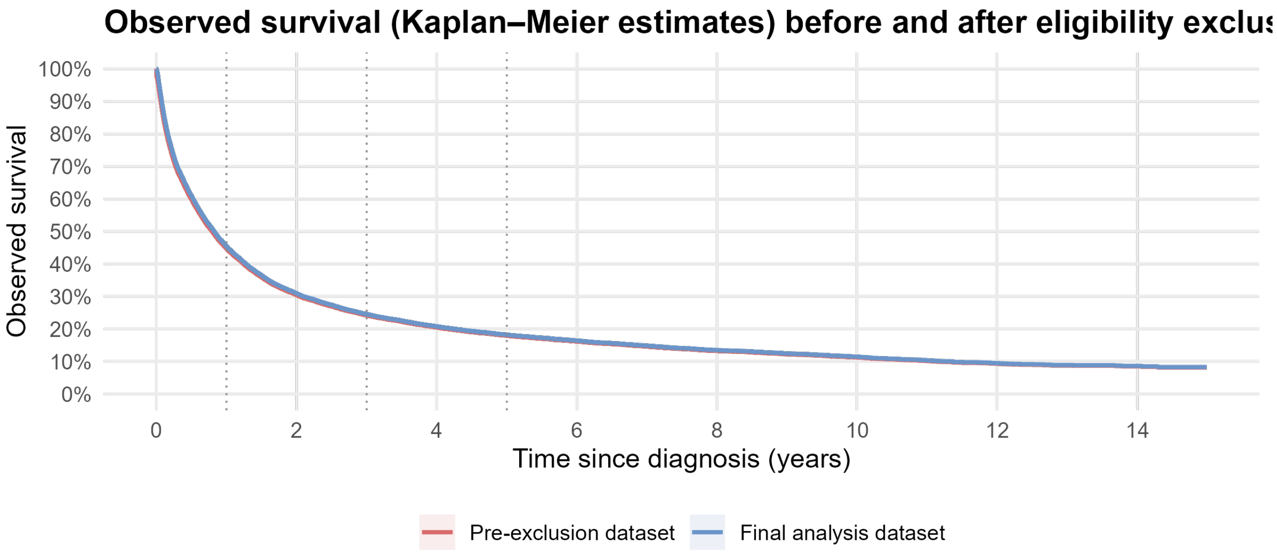

Figure 4: Observed survival before and after application of eligibility criteria. Kaplan–Meier estimates are shown for the pre-exclusion dataset and the final analysis dataset.

4 Screening results

4.1 Cohort definition

This section summarizes the empirical composition of the final analysis-ready cohort. It focuses on the size and unit structure of the dataset, the derivation of the final cohort from the prepared dataset, the final outcome-status composition, and the structural variables used to organize later screening summaries.

4.1.1 Final analysis population and unit reconciliation

The final analysis-ready dataset contains one record per eligible tumour diagnosis. The population size and the reconciliation between patient and record identifiers are summarized in Tables 10 and 11.

Table 10: Final analysis population size.

Dataset ID	Final records	Final patients	Final diseases/tumours	Unit of analysis
LUNG_SURV_2020_v2	13,973	13,973	13,973	one tumour diagnosis per patient (first primary)

Table 11: Unit reconciliation: records per patient.

Records per patient	Number of patients	Patients (%)
1 record	13,973	100%

4.1.2 Cohort derivation

The transition from the starting extracted/prepared dataset to the final analysis-ready cohort is summarized in Table 12. The same transition is shown graphically in Figure 5.

Table 12: Cohort derivation.

Dataset ID	Prepared/extracted records	Final records	Excluded records	Retained (%)	Excluded (%)
LUNG_SURV_2020_v2	14,538	13,973	565	96.1%	3.9%

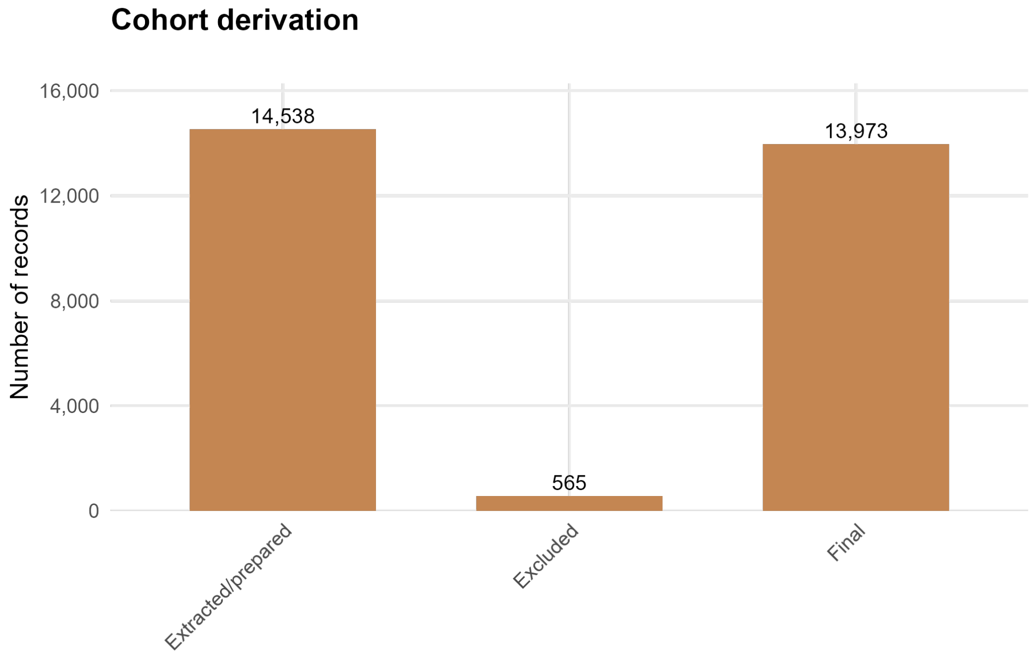

Figure 5: Cohort derivation from prepared records to the final analysis-ready dataset.

4.1.3 Outcome status composition

Outcome-status composition describes the final status of records at dataset lock. The distribution is summarized in Table 13 and shown in Figure 6.

Table 13: Outcome status composition in the final dataset.

Outcome status	Number of records	Records (%)
Death/event	12,184	87.2%
Administratively censored / event-free	1,789	12.8%

Table 14: Structural variables used for later screening summaries.

Variable	Label	Available in data	Number of distinct values	Role in screening
sex__name	Sex	Yes	2	Stratification by demographic group
diag__year	year of diagnosis	Yes	10	Stratification by diagnosis calendar year
age__group__10y	age__group__10y	Yes	8	Stratification by age group

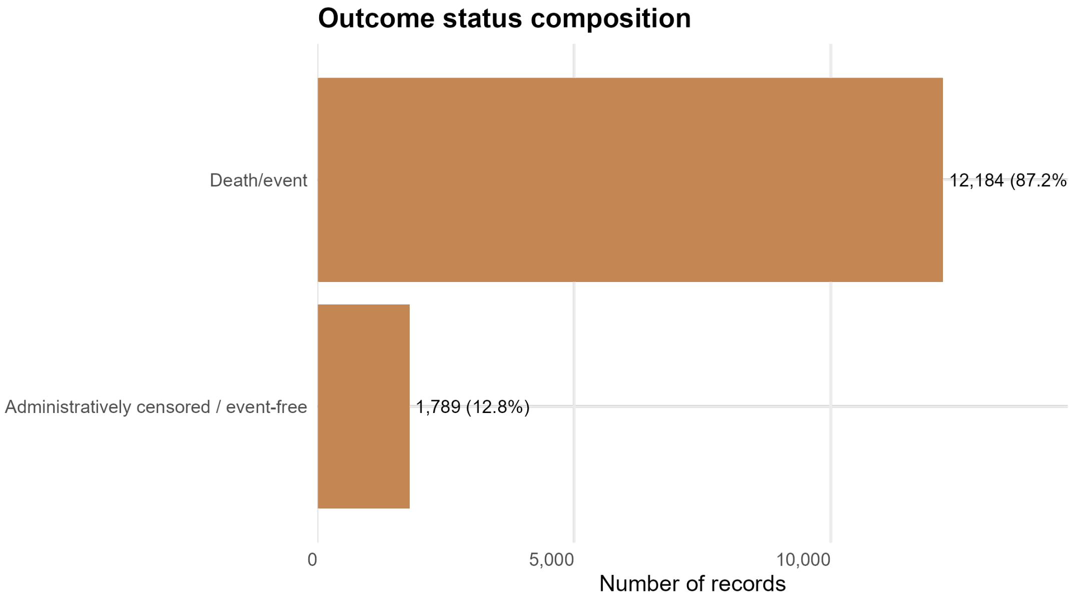

Figure 6: Outcome status composition in the final dataset.

4.1.4 Structural variables

Structural variables are analyst-defined variables used to organize stratified screening summaries. They are listed in Table 14.

4.1.5 Diagnosis-year distribution and age-period structure

This section describes how final eligible records are distributed across diagnosis years and how the age structure of the final dataset varies over calendar time. The diagnosis-year distribution is summarized in Table 15. Figure 9 displays the annual number of final eligible records together with the age-group structure by diagnosis year and sex. These outputs help identify whether changes over diagnosis years are concentrated in particular age groups and whether later temporal comparisons may require age-aware interpretation.

Table 15: Final eligible records by diagnosis year.

Diagnosis year	Records	Records (%)	Cumulative records	Cumulative records (%)
2011	1,089	8.5%	1,089	8.5%
2012	1,168	9.1%	2,257	17.6%
2013	1,179	9.2%	3,436	26.8%
2014	1,194	9.3%	4,630	36.1%
2015	1,311	10.2%	5,941	46.3%
2016	1,364	10.6%	7,305	56.9%
2017	1,345	10.5%	8,650	67.4%
2018	1,398	10.9%	10,048	78.3%
2019	1,446	11.3%	11,494	89.6%
2020	1,339	10.4%	12,833	100%

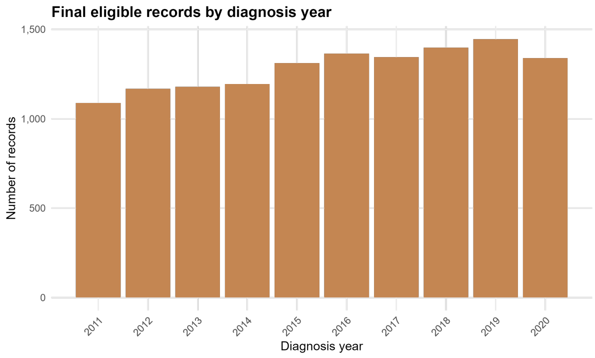

Figure 7: *
(A) Cases by diagnosis year

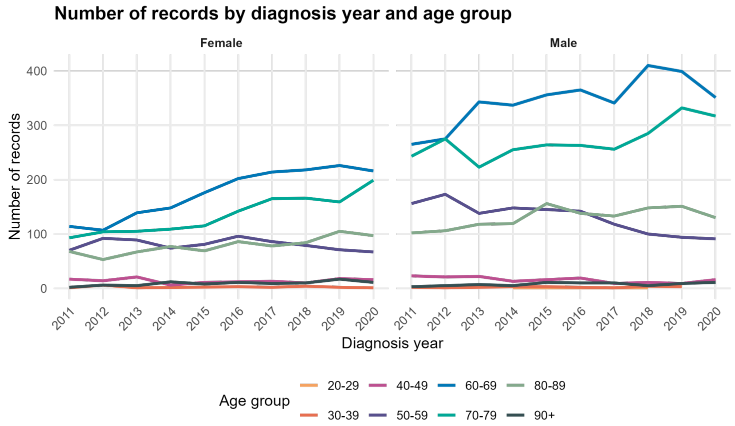

Figure 8: *
(B) Age-group structure by diagnosis year and sex

Figure 9: Cases by diagnosis year and age-period structure of the final analysis-ready dataset. Panel (A) shows the annual number of final eligible records by diagnosis year. Panel (B) shows how these records are distributed across age groups and diagnosis years, separately by sex.

4.1.6 Age, diagnosis period and cohort structure

As an optional cohort-description screen, the calendar and cohort structure of the final dataset was examined. This output describes how records are distributed across diagnosis years, age groups, and birth cohorts; temporal comparisons may be influenced by changes in the age or birth-cohort composition of the diagnosed population.

Figure 10 shows the number of records by age group. Panel A identifies which age groups contributed most to the cohort in each diagnosis year and whether increases or decreases over calendar time were concentrated in particular age groups. Panel B shows birth-cohort composition. Birth cohort was derived from diagnosis year and age at diagnosis and is interpreted descriptively as a cohort-composition screen, not as evidence of a birth-cohort effect.

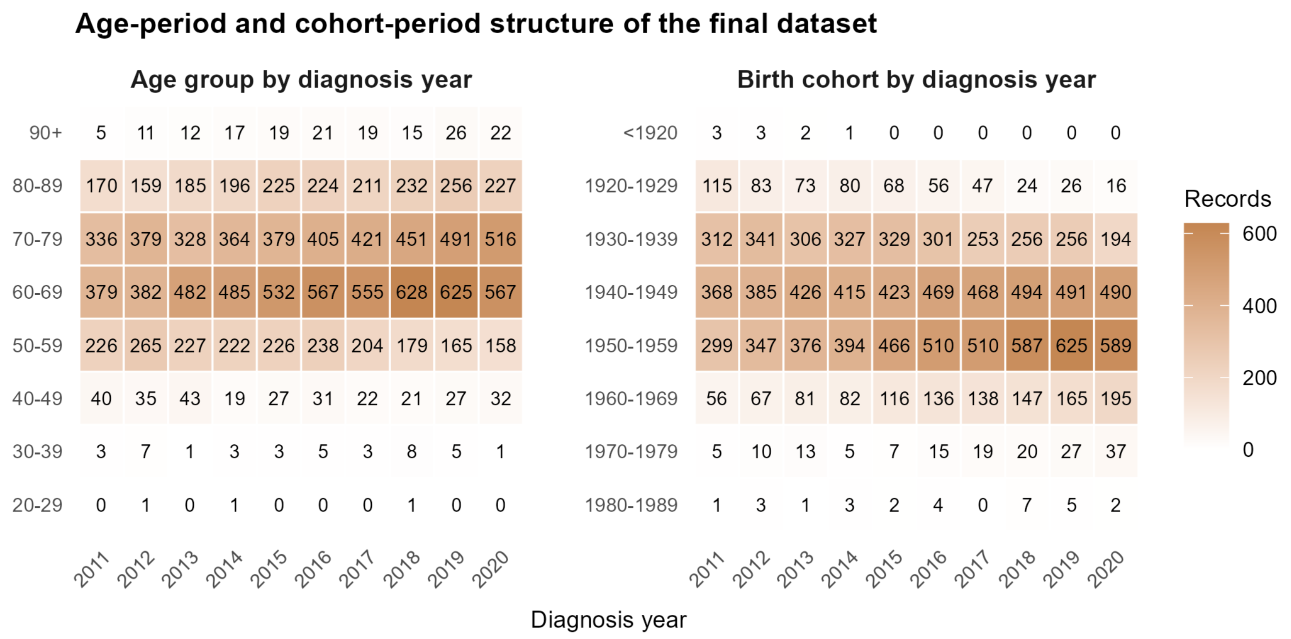

Figure 10: Age, diagnosis-year, and birth-cohort structure of the final dataset. Panel A shows the number of records by age group and diagnosis year. Panel B shows the number of records by birth cohort and diagnosis year. Birth cohort was derived from diagnosis year and age at diagnosis and is interpreted descriptively as cohort composition, not as an estimated cohort effect.

4.2 Missingness and incomplete information

This section summarizes incomplete information in the final analysis-ready dataset. Only the final dataset is assessed here. Records removed during cleaning or eligibility processing are not reintroduced into this screening section.

4.2.1 Missingness and incomplete source information

Incomplete information is classified using the central missingness categories: **Observed, Technical NA, Unknown, Not applicable, Derived unknown**. In the final dataset, not all categories necessarily occur.

Table 16: Incomplete source information by variable and type.

Variable	Incomplete information type	Total records	Records	Records (%)
Finding method	Unknown	12,833	2	0%
Registry stage	Unknown	12,833	44	0.3%

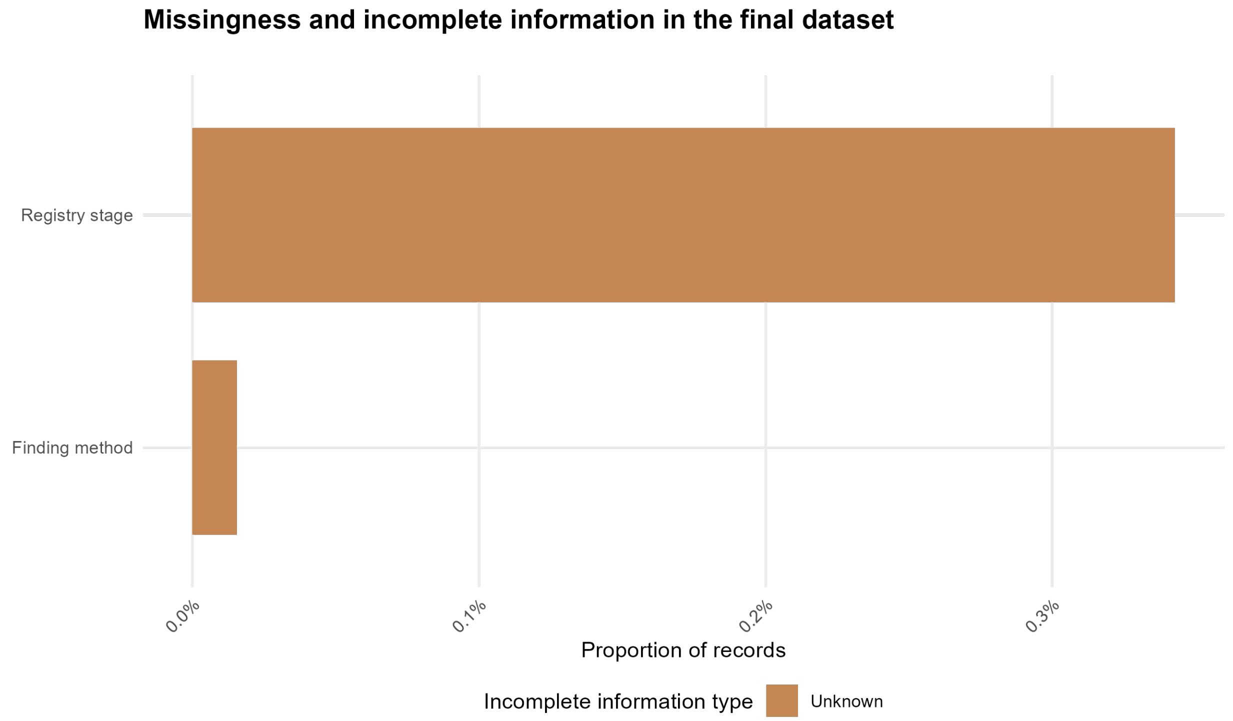

Figure 11: Missingness and incomplete information in selected source label variables and derived survival variables.

No missing values were identified in the derived survival variables in the final dataset. This means that event status, end date, survival time in days, and survival time in years were complete in the final analysis-ready dataset.

4.2.2 Structural patterns in incomplete information

Incomplete information was also summarized by structural variables. Figure 12 shows incomplete information by diagnosis year, age group, and sex. These plots are descriptive and are intended to show whether incomplete information is concentrated in particular structural subgroups.

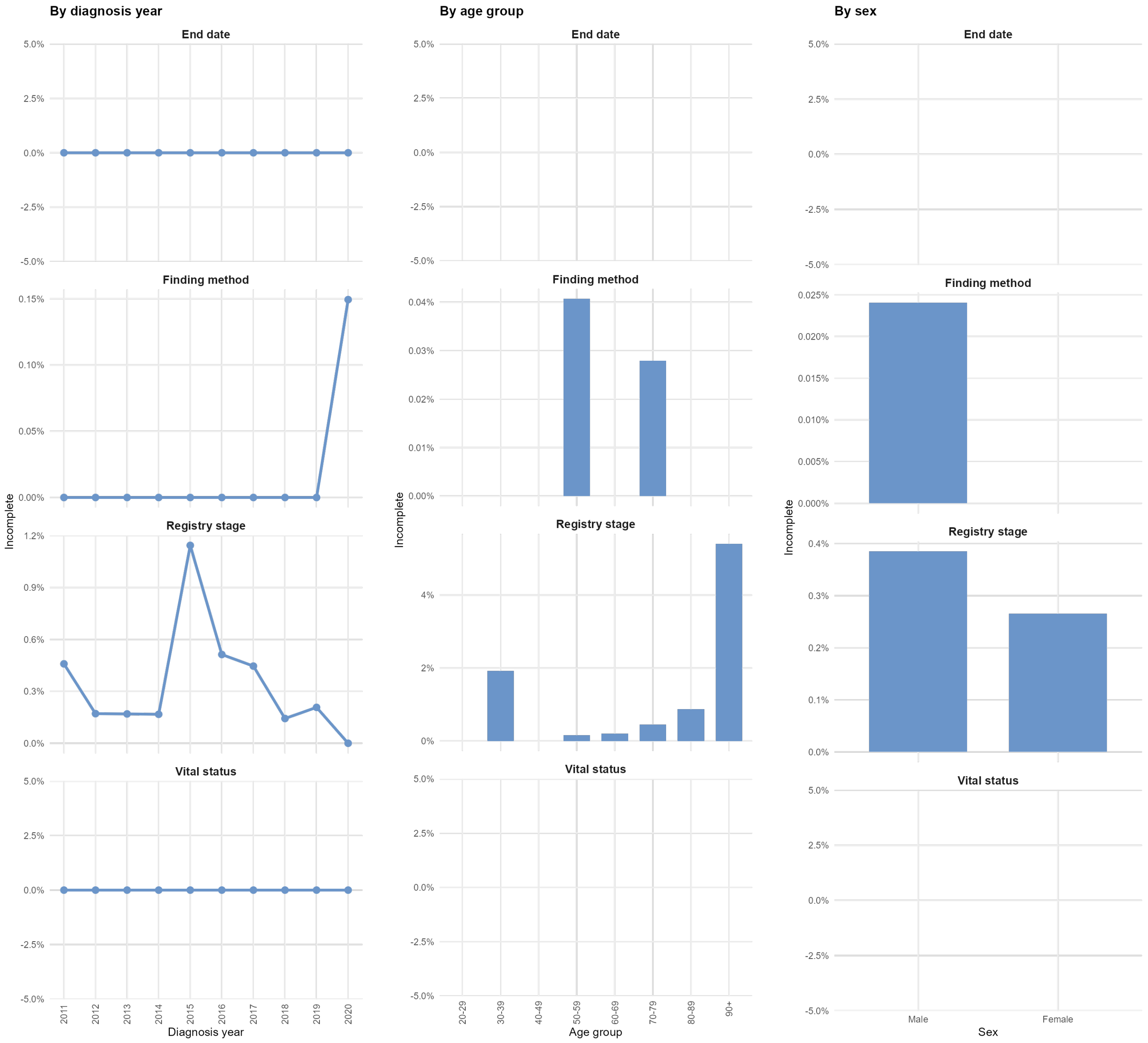

Figure 12: Incomplete information by diagnosis year, age group, and sex. Each panel uses the final analysis-ready dataset only.

4.2.3 Loss to follow-up

Loss to follow-up represents records with unknown vital status and therefore incomplete follow-up information. The table below shows the distribution of such records across age groups and sex. Percentages are calculated within each age-sex group, so each cell gives the number of records lost to follow-up and the percentage among all records in that subgroup.

No records classified as lost to follow-up were present in the final analysis-ready dataset. Therefore, no loss-to-follow-up table is shown for this item.

4.2.4 Cause-of-death-report ascertainment

Cause-of-death-report ascertainment was defined using finding method value **Cause-of-death report**. This item is handled separately from missingness because it describes ascertainment context rather than an unknown value.

No records with cause-of-death-report ascertainment were present in the final analysis-ready dataset. Therefore, no table or figure is shown for this item.

4.3 Univariate distributions

This section summarizes the marginal distributions of key variables in the final analysis-ready dataset. Numerical variables are summarized using standard descriptive statistics and selected histograms. Categorical variables are summarized using frequency tables with counts and percentages. For detailed ICD-O-3 morphology, the full distribution contains many distinct values, so the report-facing table shows the 15 most frequent morphology labels and groups the remaining values as Other. The full detailed morphology distribution is retained in the output files for traceability.

4.3.1 Numerical variables

Numerical variables describe age at diagnosis, diagnosis year, and observed follow-up or survival time. Table 17 gives descriptive statistics for these variables. Age and survival time distributions are shown in Figure 15.

Table 17: Numerical variable distributions.

Variable	Total records	Non-missing	Missing	Missing (%)	Mean	SD	Minimum	Q1	Median	Q3	Maximum
Age at diagnosis	13,973	13,973	0	0%	68.23	10.03	22.41	61.26	67.73	75.66	94.89
Diagnosis year	13,973	13,973	0	0%	2015.77	2.84	2011	2013	2016	2018	2020
Survival time, days	13,973	13,973	0	0%	832.23	1143.19	1	81	301	1062	5475
Survival time, years	13,973	13,973	0	0%	2.28	3.13	0	0.22	0.82	2.91	14.99

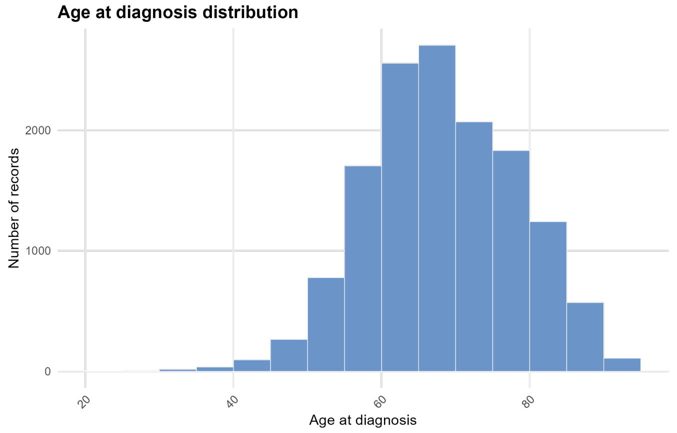

Figure 13: *
(A) Age at diagnosis

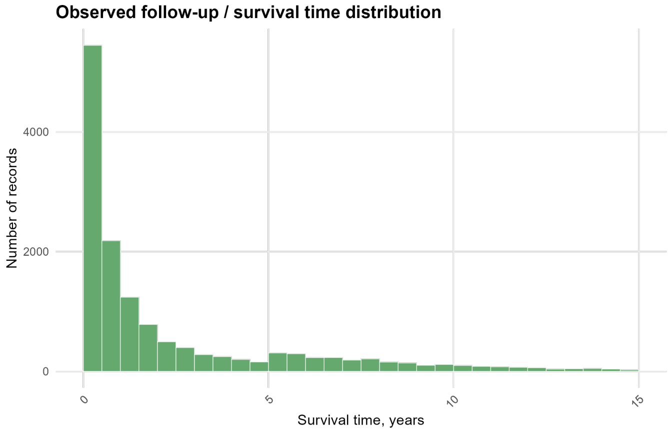

Figure 14: *
(B) Observed follow-up / survival time

Figure 15: Numerical distributions in the final dataset.

Table 18: Frequency distribution for sex.

Category	Records	Records (%)
Male	9,017	64.5%
Female	4,956	35.5%

Table 19: Frequency distribution for vital status.

Category	Records	Records (%)
Dead	12,259	87.7%
Alive	1,714	12.3%

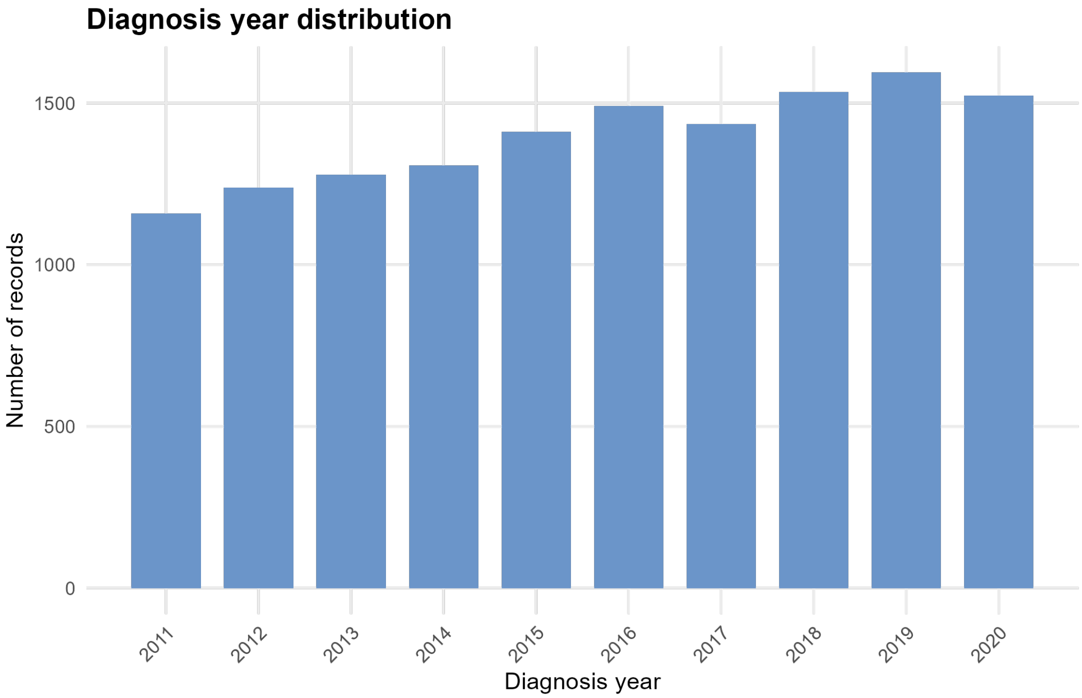

Figure 16: Diagnosis year distribution in the final dataset.

4.3.2 Categorical frequency tables

Categorical variables are summarized using full frequency tables for the report-facing label variables. Code-variable frequency tables are also saved in the output folder for traceability, but the main report emphasizes readable label variables. Detailed morphology is shown as the 15 most frequent labels plus Other in the histology subsection below.

Table 20: Frequency distribution for registry stage.

Category	Records	Records (%)
Metastatic	7,381	52.8%
Regional	4,041	28.9%
Localized	2,460	17.6%
No data	91	0.7%

Table 21: Frequency distribution for event status.

Category	Records	Records (%)
Event	12,184	87.2%
Censored	1,789	12.8%

Table 22: Frequency distribution for ICD-10 site.

Category	Records	Records (%)
Upper lobe, bronchus or lung	7,256	51.9%
Lower lobe, bronchus or lung	4,141	29.6%
Bronchus or lung, unspecified	945	6.8%
Middle lobe, bronchus or lung	789	5.7%
Main bronchus	608	4.3%
Overlapping lesion of bronchus and lung	205	1.5%
Malignant neoplasm of trachea	29	0.2%

Table 23: Frequency distribution for diagnosis basis.

Category	Records	Records (%)
Histology of primary tumour	10,056	72%
Cytology	1,953	14%
Other diagnostic examinations	1,202	8.6%
Histology of metastasis	753	5.4%
Clinical	9	0.1%

Table 24: Frequency distribution for finding method.

Category	Records	Records (%)
Clinical signs	12,670	90.7%
Other	1,301	9.3%
Unknown	2	0%

Table 25: Frequency distribution for grouped histology.

Category	Records	Records (%)
Adenocarcinoma	5,587	40%
Squamous cell carcinoma	3,357	24%
Small cell carcinoma	2,059	14.7%
Carcinoma / neoplasm NOS	1,411	10.1%
Other specified morphology	1,269	9.1%
Large cell / undifferentiated carcinoma	290	2.1%

4.3.3 Categorical distribution figures

Figures 19, 22, 25, and 28 show selected categorical distributions. For morphology, grouped histology is shown because detailed ICD-O-3 morphology has many distinct values.

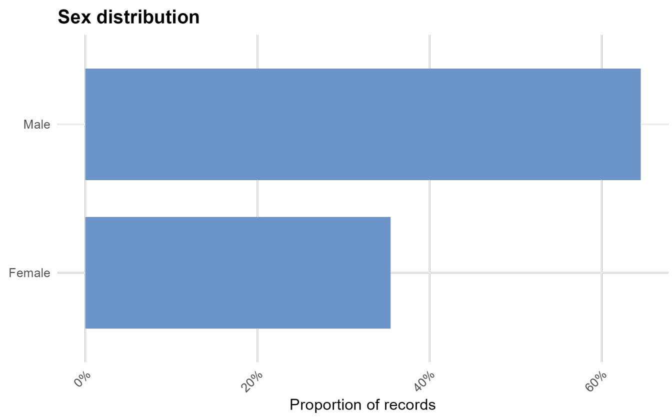

Figure 17: *
(A) Sex

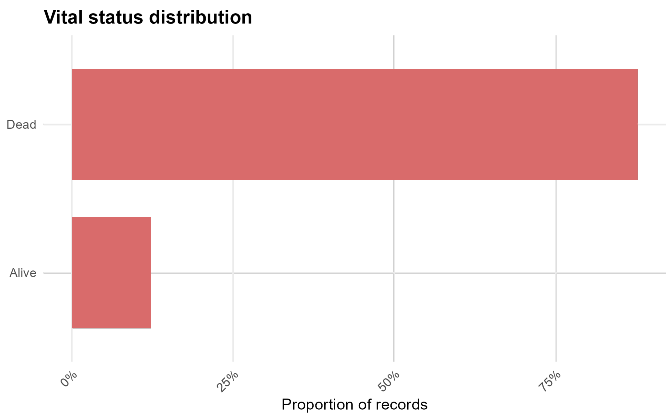

Figure 18: *
(B) Vital status

Figure 19: Sex and vital status distributions.

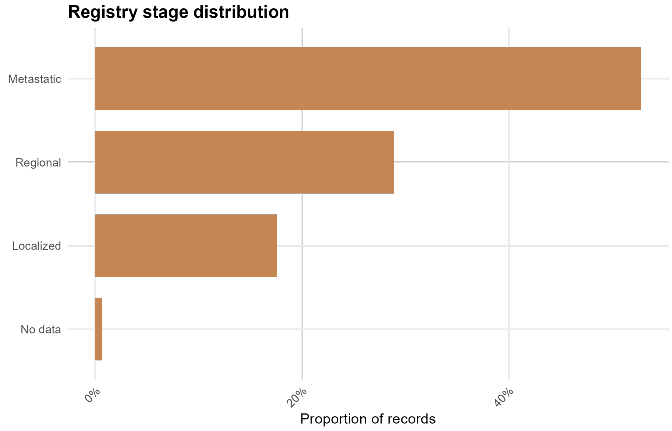

Figure 20: *
(A) Registry stage

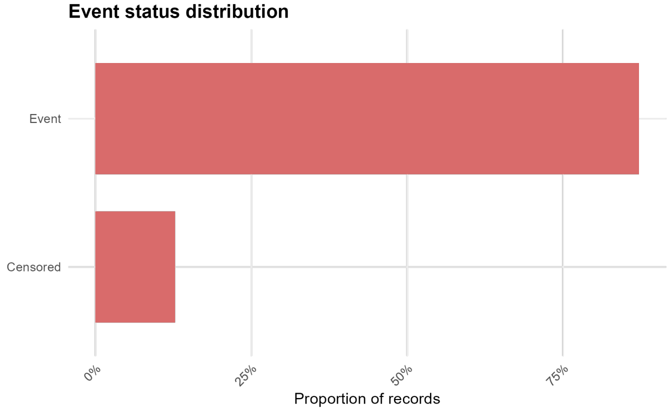

Figure 21: *
(B) Event status

Figure 22: Registry stage and event status distributions.

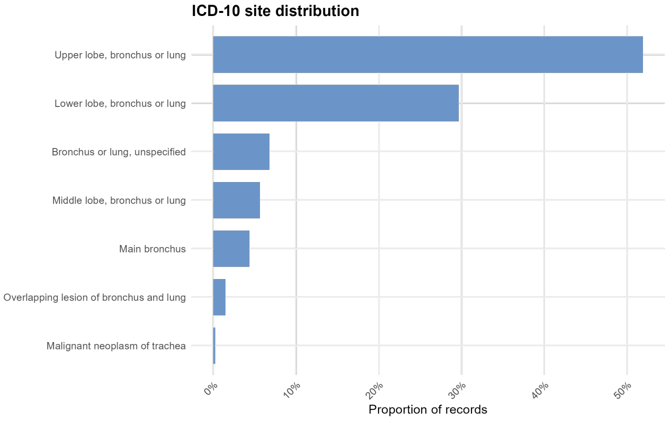

Figure 23: *
(A) ICD-10 site

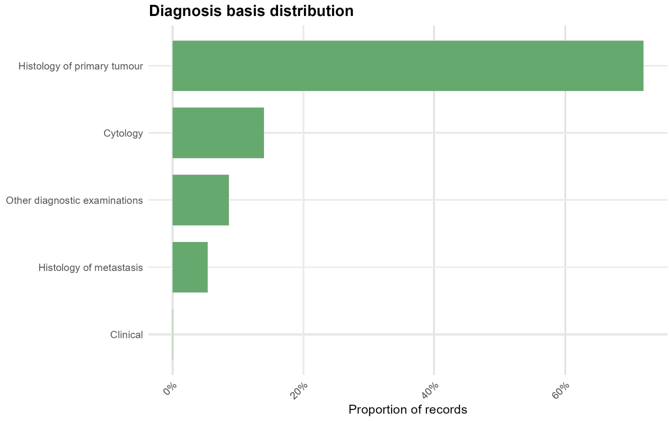

Figure 24: *
(B) Diagnosis basis

Figure 25: Cancer site and diagnosis basis distributions.

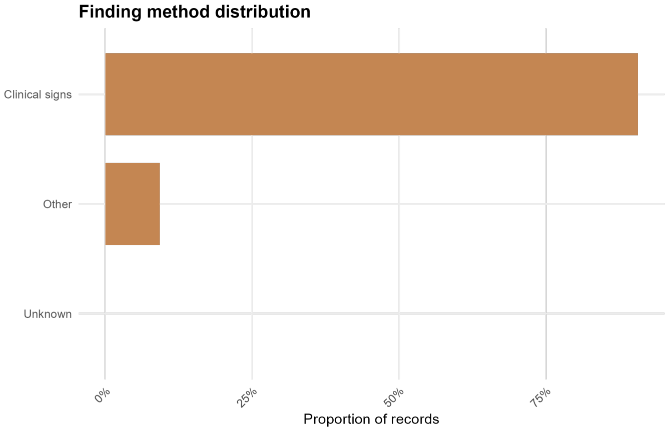

Figure 26: *
(A) Finding method

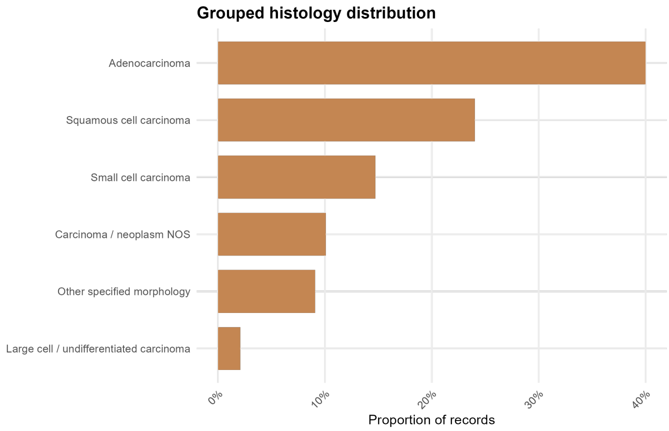

Figure 27: *
(B) Grouped histology

Figure 28: Finding method and grouped histology distributions.

4.3.4 Histology grouping and detailed morphology

Detailed ICD-O-3 morphology contains many distinct values. For graphical reporting, morphology codes were grouped into broader histology groups. The grouping is shown in Table 26. The report-facing detailed morphology table shows the 15 most frequent morphology labels and groups all remaining labels as Other. The full detailed morphology distribution is retained in the output table `screening_6_3_histology_detailed_distribution.csv`.

The full detailed morphology distribution is available in the output table `screening_6_3_histology_detailed_distrib`[illegible] Because many morphology labels are rare, the full table is more suitable for audit and traceability than for direct graphical display in the report.

Table 26: Histology grouping rules used for graphical summaries.

Histology group	ICD-O-3 morphology codes used
Adenocarcinoma	8140, 8250, 8255, 8260, 8480, 8550, 8574
Squamous cell carcinoma	8070, 8071, 8072, 8073, 8083, 8084
Small cell carcinoma	8041, 8042, 8043, 8044, 8045
Large cell / undifferentiated carcinoma	8012, 8013, 8022, 8030, 8031, 8032, 8033, 8035, 8310
Carcinoma / neoplasm NOS	8000, 8001, 8010, 8011, 8020, 8021
Other specified morphology	All other non-missing ICD-O-3 morphology codes
Missing / unknown	Missing or blank ICD-O-3 morphology code

Table 27: Detailed ICD-O-3 morphology frequency table: 15 most frequent labels and Other.

Category	Records	Records (%)
Adenocarcinoma, NOS	5,247	37.5%
Squamous cell carcinoma, NOS	2,677	19.2%
Small cell carcinoma, NOS	2,009	14.4%
Neoplasm	1,224	8.8%
Non-small cell carcinoma	819	5.9%
Squamous cell carcinoma, keratinizing, NOS	448	3.2%
Squamous cell carcinoma, large cell, nonkeratinizing, NOS	218	1.6%
Neuroendocrine tumor, NOS	179	1.3%
Carcinoma, NOS	176	1.3%
Mucinous adenocarcinoma	146	1%
Large cell neuroendocrine carcinoma	118	0.8%
Adenocarcinoma with mixed subtypes	87	0.6%
Large cell carcinoma, NOS	75	0.5%
Pleomorphic carcinoma	68	0.5%
Adenosquamous carcinoma	61	0.4%
Other	421	3%

4.4 Multivariate screening

This section summarizes selected multivariate data properties in the final analysis-ready dataset. The purpose is descriptive: to examine whether key clinical and outcome variables vary across structural variables. The structural variables used here are sex, 10-year age group, and diagnosis year. The outputs are intended to support interpretation of subgroup analyses and to identify patterns that may require attention in later modelling or reporting.

4.4.1 Key clinical variables by structural variables

The first set of multivariate checks examines whether registry stage, grouped histology, diagnosis basis, finding method, and event status differ by sex, age group, and diagnosis year. Each plot shows the distribution of one target variable within the structural groups. These summaries are descriptive and are not intended as formal hypothesis tests.

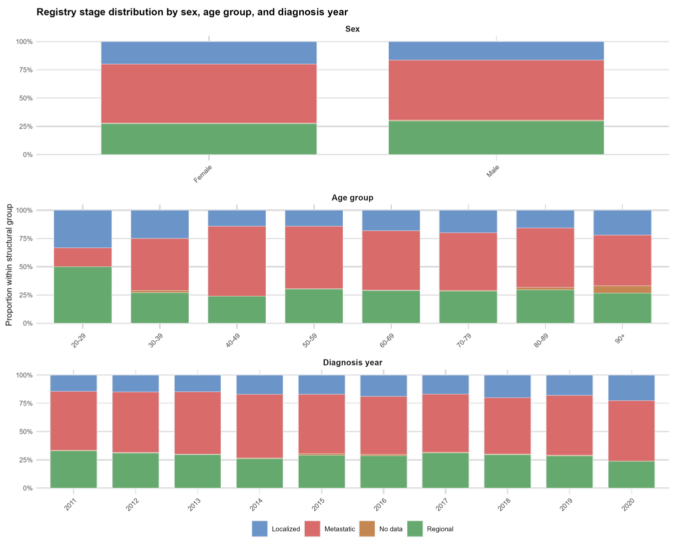

Figure 29: *
(A) Registry stage

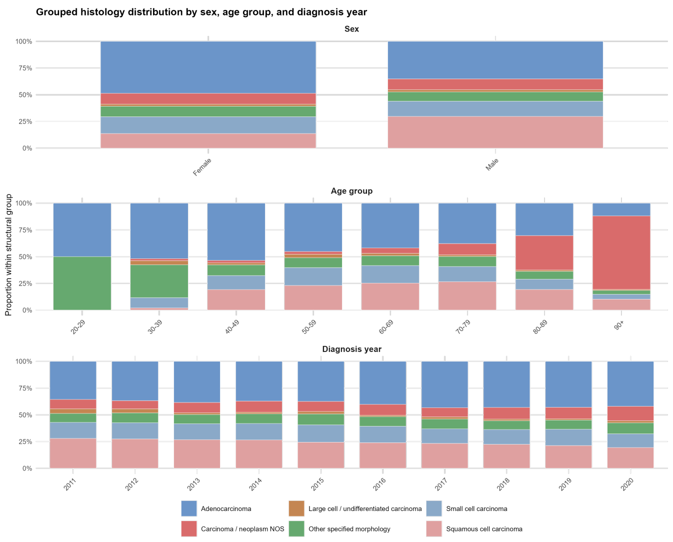

Figure 30: *
(B) Grouped histology

Figure 31: Registry stage and grouped histology by sex, age group, and diagnosis year.

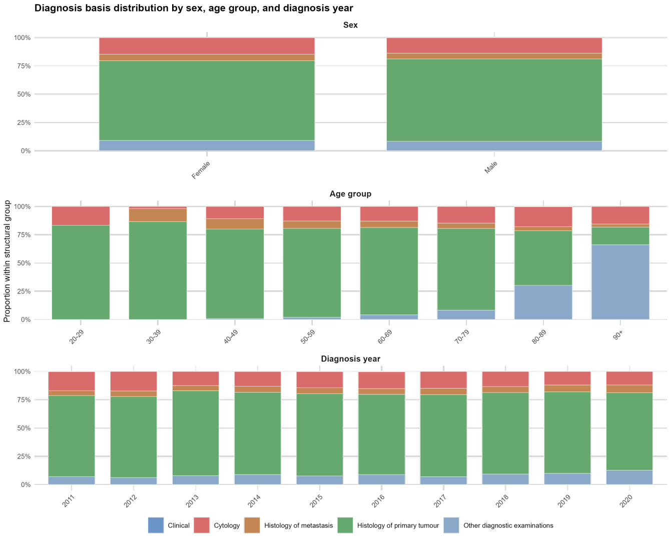

Figure 32: *
(A) Diagnosis basis

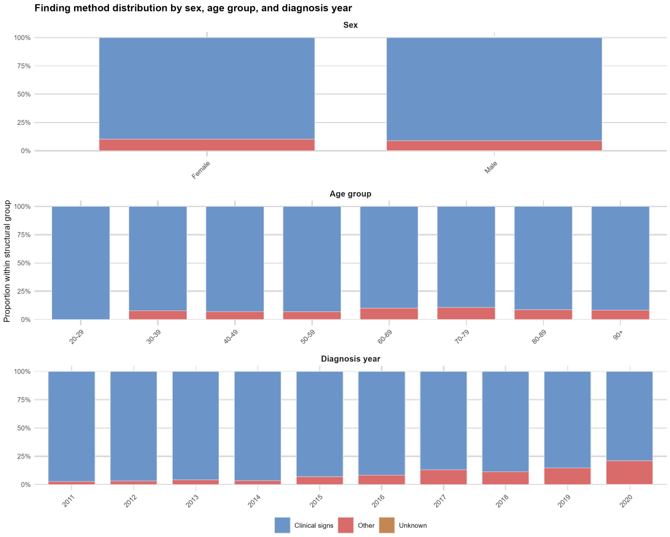

Figure 33: *
(B) Finding method

Figure 34: Diagnosis basis and finding method by sex, age group, and diagnosis year.

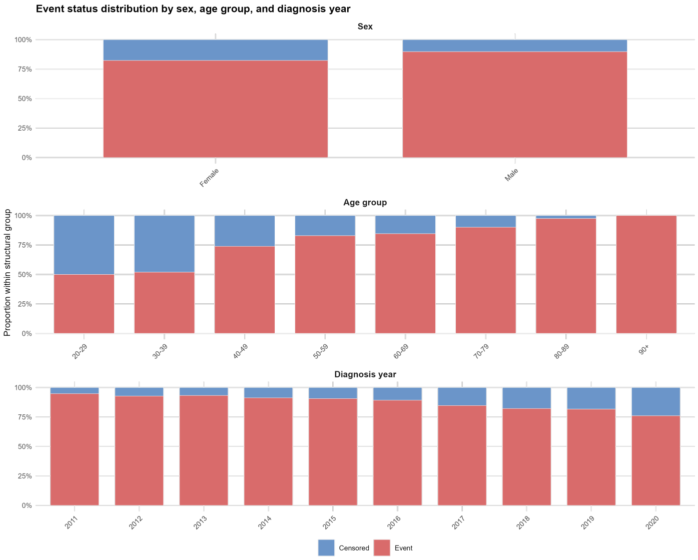

Figure 35: Event status by sex, age group, and diagnosis year.

4.4.2 Survival time by structural variables

Observed survival time was also summarized across structural groups. The aim is to identify broad differences in follow-up depth across sex, age groups, and calendar time. This is a descriptive screening output and should not be interpreted as a formal comparison of survival between groups.

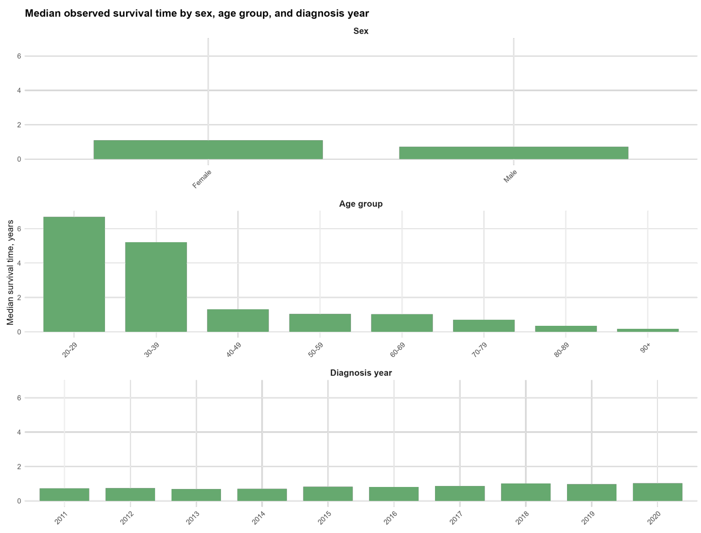

Figure 36: Median observed survival time across structural variables.

Table 28: Observed survival time by structural variables.

Structural variable	Structural value	Records	Non-missing survival time	Mean survival time	SD	Median survival time	Q1	Q3
Sex	Male	9,018	9,018	2.02	2.95	0.71	0.2	2.31
Sex	Female	4,956	4,956	2.74	3.39	1.1	0.29	4.44
Age group	20-29	6	6	6.7	5.3	6.69	2.25	10.84
Age group	30-39	52	52	5.34	4.39	5.22	0.9	8.04
Age group	40-49	360	360	3.53	4.22	1.31	0.48	5.74
Age group	50-59	2,478	2,478	2.86	3.67	1.05	0.33	4.15
Age group	60-69	5,260	5,260	2.57	3.23	1.03	0.29	3.92
Age group	70-79	3,898	3,898	1.97	2.76	0.7	0.18	2.44
Age group	80-89	1,811	1,811	1.05	1.74	0.36	0.11	1.12
Age group	90+	109	109	0.5	0.89	0.17	0.07	0.64
Diagnosis year	2011	1,159	1,159	2.19	3.69	0.73	0.22	1.94
Diagnosis year	2012	1,239	1,239	2.45	3.88	0.75	0.22	2.29
Diagnosis year	2013	1,278	1,278	2.15	3.44	0.69	0.21	1.93
Diagnosis year	2014	1,307	1,307	2.31	3.5	0.7	0.2	2.29
Diagnosis year	2015	1,411	1,411	2.3	3.26	0.83	0.23	2.64
Diagnosis year	2016	1,491	1,491	2.25	3.05	0.8	0.23	2.68
Diagnosis year	2017	1,435	1,435	2.46	3.06	0.86	0.24	3.61
Diagnosis year	2018	1,535	1,535	2.46	2.83	1.01	0.23	4.22
Diagnosis year	2019	1,595	1,595	2.13	2.42	0.97	0.22	3.67
Diagnosis year	2020	1,524	1,524	2.1	2.19	1.03	0.23	4.96

4.5 Survival-specific aspects

This section summarizes basic survival and follow-up information in the final analysis-ready dataset and presents sex-specific feasibility outputs for subgroup-specific survival estimation.

4.5.1 Basic survival and follow-up summary

The following tables summarize the basic survival outcome structure of the final dataset.

Table 29: Basic survival and follow-up summary.

Records	Events	Censored	Missing event status	Events (%)	Censored (%)	Median survival time, years
13,973	12,184	1,789	0	87.2%	12.8%	0.82

Table 30: Event status by sex.

Sex	Event status	Records	Records (%)
Female	Censored	873	17.6%
Female	Event	4,083	82.4%
Male	Censored	916	10.2%
Male	Event	8,101	89.8%

4.5.2 Sex-specific age-group support and Kaplan–Meier curves

The figures below summarize age-specific support for selected survival estimates and the corresponding Kaplan–Meier curves, separately for women and men. In the heatmap, each cell shows the number of cases diagnosed early enough to allow the selected follow-up time, followed by the number still at risk at that time point. A subgroup was considered supported when both counts were at least 10. The Kaplan–Meier panels show observed survival curves only for age groups that met the support criteria for at least one survival horizon. Dashed vertical lines indicate the longest supported survival time for each age group.

Unsupported age groups are summarized below when present. These groups are not shown as subgroup-specific Kaplan–Meier curves and should not be used for survival estimates at unsupported horizons without regrouping or qualification.

For women, the unsupported age groups were: 20-29; 90+.

For men, the unsupported age groups were: 20-29.

Table 31: Maximum supported survival horizon overall and by sex.

Subgroup structure	Subgroup	Maximum supported by baseline follow-up	Maximum supported by risk set
overall	All cases	10-year survival	10-year survival
sex	Female	10-year survival	10-year survival
sex	Male	10-year survival	10-year survival

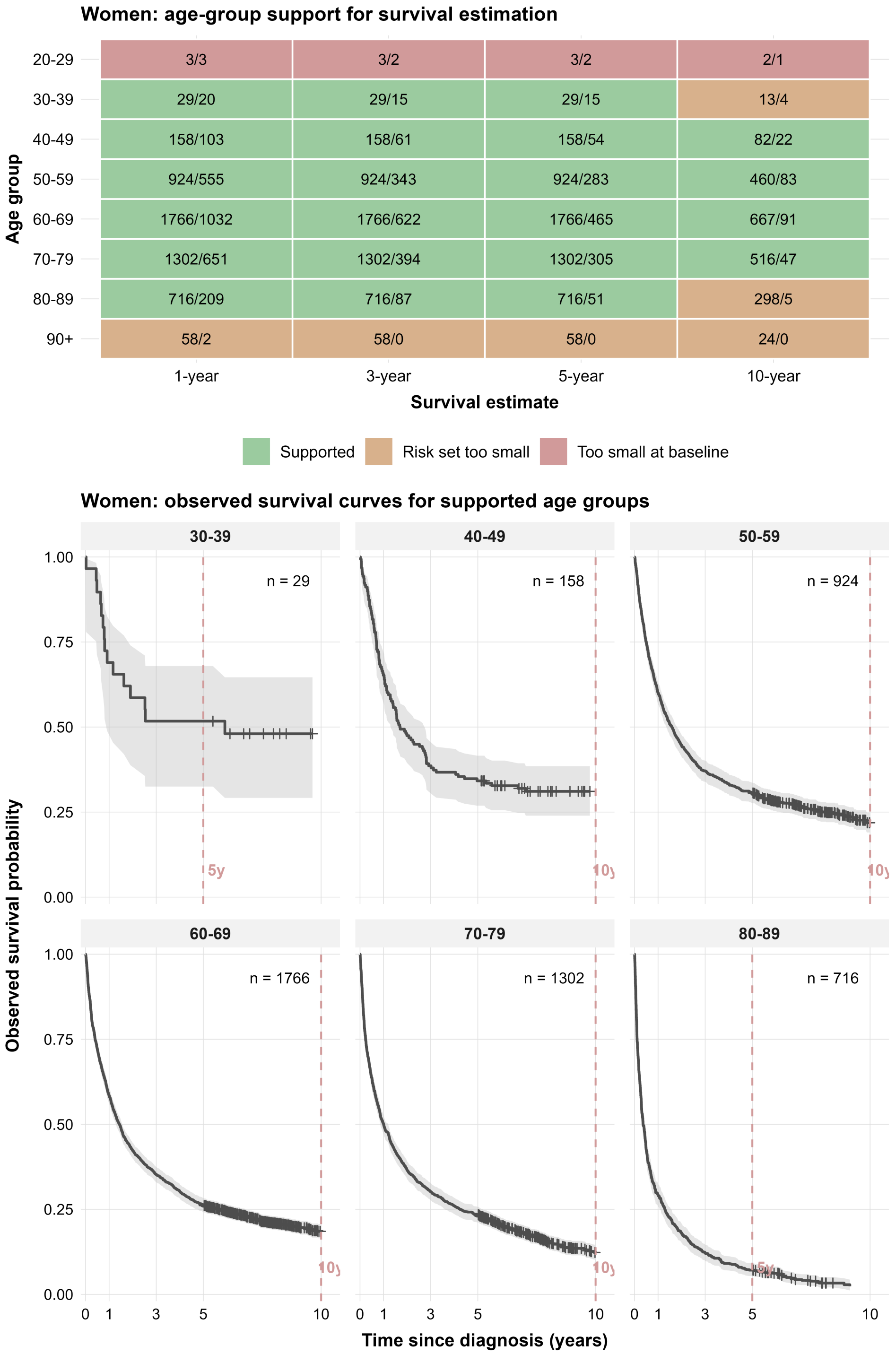

Figure 37: Age-group support for selected survival estimates and observed Kaplan–Meier curves for women. The heatmap shows, for each survival horizon, the number of cases diagnosed early enough to allow that follow-up time and the number still at risk at that time point. Kaplan–Meier curves are shown only for supported age groups.

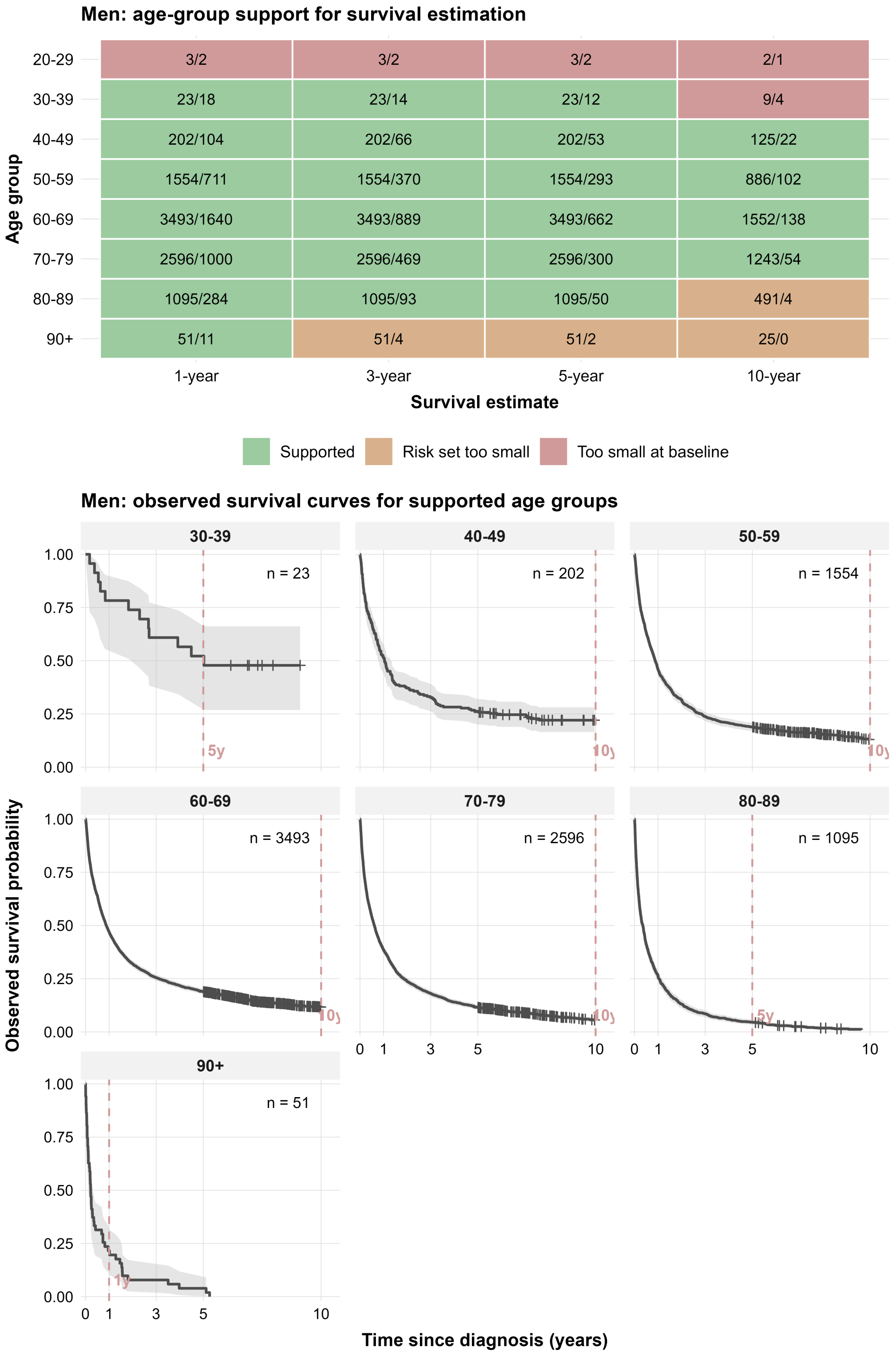

Figure 38: Age-group support for selected survival estimates and observed Kaplan–Meier curves for men. The heatmap shows the number of cases diagnosed early enough to allow that follow-up time and the number still at risk at that time point. Kaplan–Meier curves are shown only for supported age groups.

Together, these sex-specific figures document which age subgroups can support the selected survival estimates. They are intended to guide which subgroup survival estimates should be reported directly, which should be restricted to shorter horizons, and which may require regrouping or omission.

4.5.3 Preparedness for net survival

This section assesses whether the final dataset contains the core structural information required for possible net survival analysis. It focuses on the availability of variables needed for linkage to population mortality tables and on eligibility restrictions relevant to net survival.

4.5.3.1 Required variables The core variables assessed here are age at diagnosis, sex, diagnosis date, survival time, and event status. These variables are required to define follow-up and to link each record to the appropriate population mortality table by age, sex, and calendar time.

Table 32: Availability of core variables required for net survival analysis.

Variable	Present in final dataset	Missing records	Missing records (%)
age_diag	Yes	0	0%
sex_code	Yes	0	0%
diag_date	Yes	0	0%
surv_time_days	Yes	0	0%
event	Yes	0	0%

4.5.3.2 Net-survival-related eligibility rules

Table 33: Net-survival-related eligibility and preparation rules.

Rule ID	Rule name	Executed	Records affected	Comment
R22	Completeness for net survival	yes	0	Required variables for life-table linkage checked.
R23	Maximum age restriction for net survival	yes	15	Applied from rule_parameters: age_max_net = 95.
R24	Valid sex for net survival	not_active	0	Not required for this rule set.
R27	Diagnosis date precision restriction	yes	28	Excluded records where only diagnosis year is valid; month-year and cor
R28	Known administrative unit restriction	yes	62	Excluded records with administrative unit NEZNANO.

4.5.3.3 Brief assessment The final dataset contains complete information for the core variables required for net survival analysis. In addition, the active rule set includes eligibility checks relevant to expected-mortality linkage: completeness for net survival, age restrictions, valid sex, diagnosis-date precision, and administrative-unit availability where applicable.

Subgroup-specific feasibility should be interpreted together with the follow-up support and risk-set screening outputs reported earlier in this document.

5 Final analysis-ready dataset

This section summarizes the final researcher-facing dataset produced by the IDA workflow. The dataset shown here is the final analysis-ready version after rule-based preparation, eligibility decisions, derivations, and variable selection according to the variable catalogue.

5.1 Final dataset dimensions

Table 34: Dimensions of the final researcher-facing dataset.

Number of records	Number of variables
13,973	25

5.2 Variables included in the final dataset

The table below presents the variable catalogue for the final dataset. To keep the report readable in portrait layout, the catalogue is split into two tables: one describing the variables and one documenting coding, missingness, and derivation details.

Table 35: Variable catalogue: variable description

Variable	Description	Type
study__patient__id	unique patient identifier	ID
study__disease__id	unique tumour/disease record identifier	ID
sex__code	sex	categorical
age__diag	age at diagnosis (years)	numeric
diag__date	date of diagnosis	date
diag__year	year of diagnosis	integer
mkb10__code	ICD-10 topography code	categorical
mkb10__3char	ICD-10 3-character site	categorical
histology__code	morphology / histology code	categorical
death__date	date of death	date
event	event indicator for overall survival	binary
end__date	end of follow-up	date
surv__time__days	survival time in days	numeric
surv__time__years	survival time in years	numeric
registry__stage__code	registry stage	categorical
stage__definition__code	stage definition / staging system version indicator	categorical

Table 36: Variable catalogue: coding, missingness, and derivation

Variable	Coding values	Missing definition	Derivation
study__patient__id	unique numeric ID	none	source
study__disease__id	unique numeric ID	none	source
sex__code	1=male, 2=female	other/unknown codes possible	source
age__diag	years	NA=missing	source
diag__date	YYYY-MM-DD	NA=invalid/missing	source
diag__year	2011–2020	NA=missing	source / from diag__date
mkb10__code	C33, C34, subcodes	NA=missing	source
mkb10__3char	C33 / C34	NA=missing	source / from mkb10__code
histology__code	ICD-O morphology code	NA=missing	source
death__date	YYYY-MM-DD	NA=alive or unavailable	source
event	1=death before or on follow-up end; 0=alive/censored at follow-up end	NA=unknown vital status / cannot determine	derived
end__date	death date or administrative censoring date	NA=cannot determine	derived
surv__time__days	0	NA=missing	derived
surv__time__years	continuous	NA=missing	derived
registry__stage__code	registry-specific stage codes	NA=missing/unknown	source
stage__definition__code	registry-specific definition codes	NA=missing/unknown	source

Table 37: Locking metadata for the final dataset.

dataset_id	locked_at	dataset_status	rule_set_id
LUNG_SURV_2020_v2	2026-07-09 21:54:51.154494	analysis-ready demo dataset	RS_SURV_LUNG_DEMO_v2

Table 39: R session information.

Element	Value
R version	4.4.1
Platform	x86_64-w64-mingw32/x64
Operating system	Windows 11 x64 (build 22621)
Time zone	Europe/Ljubljana
Locale	Slovenian_Slovenia.utf8

6 Reproducibility and locking

6.1 Dataset status

This report describes a prepared dataset intended to function as a fixed, analysis-ready version for the current demonstrative use case.

The following properties support reproducibility: a clear dataset definition, a defined rule set, structured rule-execution logging, structured screening summaries, a final researcher-facing dataset consistent with the variable catalogue, recorded locking metadata for the final dataset, and defined links between the final dataset and related outputs.

6.2 Locking metadata recorded for this dataset

The final analysis-ready dataset was assigned a unique locked dataset identifier and recorded together with rule-set and versioning information.

The following elements were recorded at the moment of locking: final dataset identifier, rule set identifier, lock timestamp, number of input, final, and excluded records, analyst responsible, file paths to linked outputs, and checksum of the final dataset.

6.3 Links to outputs related to the final dataset

The outputs below identify the files linked to this dataset version. For readability, file paths are shown from the project directory level onward.

Table 38: Links to outputs related to the final dataset.

Dataset ID	Output type	Path
LUNG_SURV_2020_v2	data_final	survival_dataset_demo/outputs/LUNG_SURV_2020_v2/rds/data_final.rds
LUNG_SURV_2020_v2	data_cleaned	survival_dataset_demo/outputs/LUNG_SURV_2020_v2/rds/data_cleaned.rds
LUNG_SURV_2020_v2	rule_log	survival_dataset_demo/outputs/LUNG_SURV_2020_v2/logs/rule_execution_log.csv
LUNG_SURV_2020_v2	ida_report	survival_dataset_demo/outputs/LUNG_SURV_2020_v2

7 Session information

The session information below records the R environment used to render this IDA report.

Table 40: Attached R packages used in report rendering.

Package	Version
dplyr	1.1.4
ggplot2	3.5.2
janitor	2.2.1
kableExtra	1.4.0
knitr	1.48
lubridate	1.9.3
purrr	1.0.2
readr	2.1.6
readxl	1.4.5
stringr	1.5.1
tibble	3.2.1
tidyr	1.3.1
